# A human secretome library screen reveals a role for Peptidoglycan Recognition Protein 1 in Lyme borreliosis

**DOI:** 10.1371/journal.ppat.1009030

**Published:** 2020-11-11

**Authors:** Akash Gupta, Gunjan Arora, Connor E. Rosen, Zachary Kloos, Yongguo Cao, Jiri Cerny, Andaleeb Sajid, Dieuwertje Hoornstra, Maryna Golovchenko, Natalie Rudenko, Ulrike Munderloh, Joppe W. Hovius, Carmen J. Booth, Christine Jacobs-Wagner, Noah W. Palm, Aaron M. Ring, Erol Fikrig

**Affiliations:** 1 Section of Infectious Diseases, Department of Internal Medicine, Yale University School of Medicine, New Haven, Connecticut, United States of America; 2 Department of Immunobiology, Yale University School of Medicine, New Haven, Connecticut, United States of America; 3 Microbiology Program, Yale School of Medicine, New Haven, Connecticut, United States of America; 4 Department of Clinical Veterinary Medicine, and Key Laboratory for Zoonosis Research, Ministry of Education, College of Veterinary Medicine, Jilin University, Changchun, China; 5 Faculty of Tropical AgriSciences, Czech University of Life Sciences in Prague, Prague, Czech Republic; 6 Amsterdam UMC, University of Amsterdam, Center for Experimental and Molecular Medicine, Amsterdam Infection and Immunity, Amsterdam, Netherlands; 7 Biology Centre, Institute of Parasitology Czech Academy of Sciences, Buweiss, Czech Republic; 8 Department of Entomology, University of Minnesota, St. Paul, Minnesota, United States of America; 9 Department of Comparative Medicine, Yale University School of Medicine, New Haven, Connecticut, United States of America; 10 Department of Biology, Stanford University, Stanford, California, United States of America; 11 ChEM-H Institute, Stanford University, Stanford, California, United States of America; 12 Howard Hughes Medical Institute, Chevy Chase, Maryland, United States of America; Medical College of Wisconsin, UNITED STATES

## Abstract

Lyme disease, the most common vector-borne illness in North America, is caused by the spirochete *Borrelia burgdorferi*. Infection begins in the skin following a tick bite and can spread to the hearts, joints, nervous system, and other organs. Diverse host responses influence the level of *B*. *burgdorferi* infection in mice and humans. Using a systems biology approach, we examined potential molecular interactions between human extracellular and secreted proteins and *B*. *burgdorferi*. A yeast display library expressing 1031 human extracellular proteins was probed against 36 isolates of *B*. *burgdorferi sensu lato*. We found that human Peptidoglycan Recognition Protein 1 (PGLYRP1) interacted with the vast majority of *B*. *burgdorferi* isolates. In subsequent experiments, we demonstrated that recombinant PGLYRP1 interacts with purified *B*. *burgdorferi* peptidoglycan and exhibits borreliacidal activity, suggesting that vertebrate hosts may use PGLYRP1 to identify *B*. *burgdorferi*. We examined *B*. *burgdorferi* infection in mice lacking PGLYRP1 and observed an increased spirochete burden in the heart and joints, along with splenomegaly. Mice lacking PGLYRP1 also showed signs of immune dysregulation, including lower serum IgG levels and higher levels of IFNγ, CXCL9, and CXCL10.Taken together, our findings suggest that PGLYRP1 plays a role in the host’s response to *B*. *burgdorferi* and further demonstrate the utility of expansive yeast display screening in capturing biologically relevant interactions between spirochetes and their hosts.

## Author summary

Lyme disease is the most common vector-borne illness in North America and is caused by the spirochete *Borrelia burgdorferi*. The disease starts with a tick bite that leads to a skin rash and inflammation in other organs of the body, such as hearts and joints. *B*. *burgdorferi* uses many strategies to evade detection and persist in the human host. It is important to have efficient methods to be able to identify the various components of the immune system that interact with *B*. *burgdorferi* to better understand the disease process, but few currently exist. In this study, we used a novel yeast display screening assay of over 1,000 human immune proteins probed against several isolates of *Borrelia* to uncover biologically relevant interactions for the Lyme disease pathogen. We identified Peptidoglycan Recognition Protein 1 (PGLYRP1), an innate immune protein important in defense against bacteria, as a major candidate from this screen. We validated the interaction of PGLYRP1 with *Borrelia* and were able to use PGLYRP1-deficient mice as a model to understand the role of this protein in Lyme disease pathogenesis. Our study demonstrates the potential implications of yeast screens in uncovering important host-pathogen interactions.

## Introduction

Lyme disease is the most common tick-borne illness in North America. The causative spirochete *Borrelia burgdorferi* is primarily transmitted by *Ixodes scapularis* ticks [1]. The disease in humans often begins with a pathognomonic skin rash, *erythema migrans*. Disseminated infection can lead to arthritis, carditis, and neurological symptoms, among other clinical manifestations [2,3].

The pathogenesis of Lyme disease is multifactorial, involving both bacterial components that influence virulence, dissemination and infectivity, and host factors that are responsible for inflammation and the modulation of infection [4,5]. The success of *B*. *burgdorferi* as a pathogen can be attributed, in part, to its morphology. Its outer membrane contains lipid raft-like microdomains that are associated with lipoproteins [6]. Some of the surface displayed lipoproteins are immunogenic and are involved in the pathogenesis of Lyme disease. To evade host defenses and to interact with specific host factors, *B*. *burgdorferi* alters the expression of various lipoproteins throughout its life cycle [7,8]. In addition, flagellin helps maintain the corkscrew-like shape of the spirochete and facilitate movement [9]. *B*. *burgdorferi* also possesses an atypical peptidoglycan (PG) in which the canonical lysine or meso-diaminopimelic acid residue at the third position of stem peptides is replaced by ornithine [10,11]. The PG meshwork surrounds the cytoplasmic membrane and is composed of glycan strands cross-linked by short peptides containing D- and L-amino acids [12]. In diderm bacteria, including *B*. *burgdorferi*, the outer membrane shields the PG meshwork from the external environment [13]. PG, including that of *B*. *burgdorferi* [11], can be recognized by host pattern recognition receptors and stimulate immune responses [14]. The mammalian immune response to *B*. *burgdorferi* involves both humoral and cell-mediated factors that help to control or eliminate spirochetes [15–17]. There is a critical need to identify specific host immune proteins that interact with spirochete components, including lipoproteins, flagellar proteins, peptidoglycan and glycolipids.

Traditional approaches to identify potential interactions between host immune proteins and *B*. *burgdorferi* ligands are slow and arduous, requiring overexpression of bacterial ligands and potential immune receptors in their native forms. It is imperative to develop new methods of characterizing immune interactions with the spirochete in a high-throughput manner. Systems biology approaches, such as yeast display technology, enable simultaneous screening of large numbers of candidate host proteins. Yeast display, in particular, enables facile expression of mammalian host proteins in their native conformations at high surface densities that allow detection of even low-affinity interactions [18]. In this study, we employed a recently developed technology termed BASEHIT (**BA**cterial **S**election to **E**lucidate **H**ost-microbe **I**nteractions in high **T**hroughput; Rosen et al, manuscript in preparation) to simultaneously and comprehensively assess potential molecular-level interactions between *B*. *burgdorferi* and thousands of human extracellular and secreted proteins. Through this screen, we identify Peptidoglycan Recognition Protein 1 (PGLYRP1) as a key host protein that binds the atypical peptidoglycan of *B*. *burgdorferi* and establishes that PGLYRP1 is required for optimal host responses against the pathogen.

## Results

### Identification of human host factors that interact with *B*. *burgdorferi*

**Fig 1:**
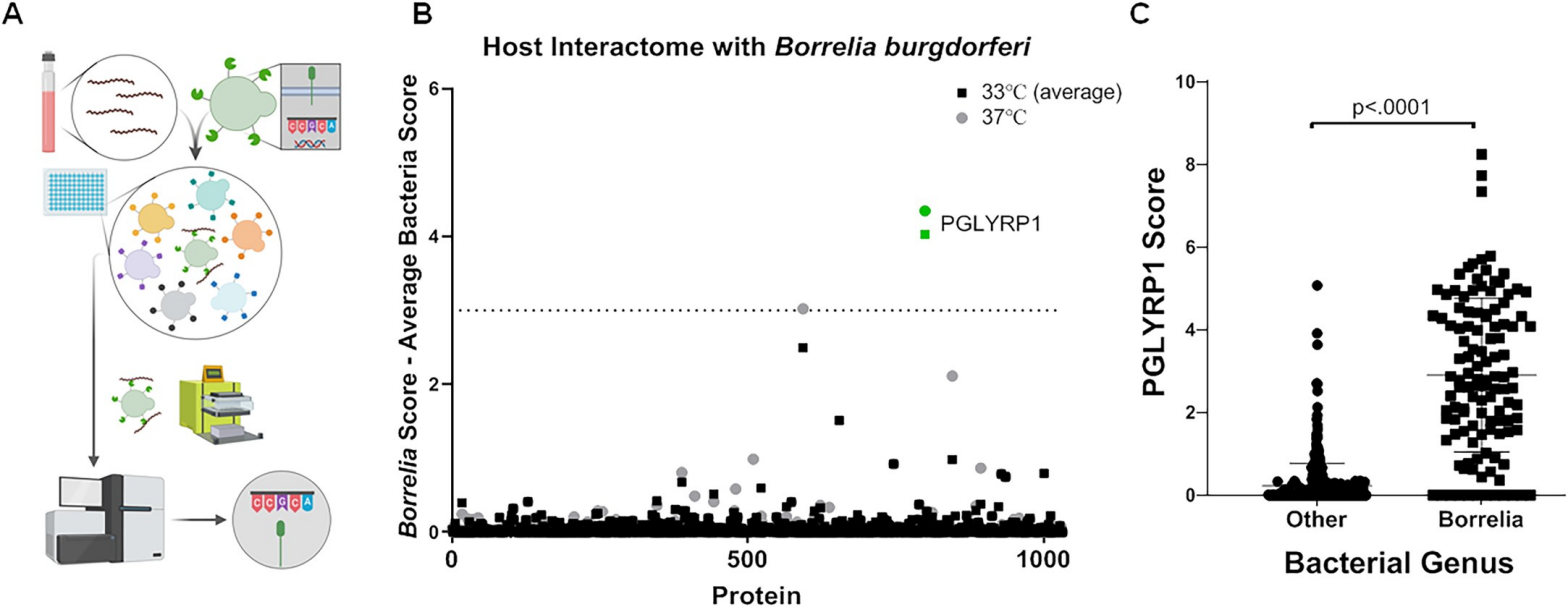
Screening with Yeast-display library. (A) Schematic of yeast-display screen- A library of yeast cells, each displaying a single human protein encoded by a uniquely bar-coded plasmid was pooled and mixed with surface biotinylated *Borrelia*. The BASEHIT library was scaled to 96-well magnetic separation format for this screen. Magnetic separation using streptavidin microbeads, followed by next-generation sequencing was used to identify yeast displaying proteins that bind to the bacterial cells. Plasmid DNA was isolated and sequenced to identify the proteins (B) Host interactions with *B*. *burgdorferi* N40- spirochete from the cultures grown at 33°C or 37°C were surface biotinylated and used for yeast selections, as described above. The data shows average scores of four independent runs of the selection on the 33°C grown sample and one run on the 37°C grown sample. The scores were normalized for diverse microbes (i.e. *Escherichia coli*, *Staphylococcus aureus*, *Bacillus subtilis*, *Shigella flexneri*, and *Bifidobacterium adolescentis*) as background correction for non-specific binding activity. (C) PGLYRP1 interaction with *Borrelia* species- Samples from 53 *Borrelia* isolates, grown at two different temperatures (when possible), were screened against the host protein library as described above for *B*. *burgdorferi*, as were 370 additional bacterial samples. The score for each gene is defined as the overall enrichment for that gene (relative to the unselected library) multiplied by the percentage of barcodes associated with the gene that enriched (defined as logFC >0). The calculated scores of *Borrelia* species were compared with those of the other bacteria. The bars represent mean ± SD and p-values were determined using a Mann-Whitney U-test.

We used a recently developed combinatorial screening technology termed BASEHIT (Rosen et al, manuscript in preparation) to identify specific human proteins that interact with *B*. *burgdorferi* and thus may be involved in pathogenesis or protection. The principle of the BASEHIT assay entails using intact, surface-biotinylated *B*. *burgdorferi* to pan a curated, genetically-barcoded yeast-display library of >1,000 human extracellular and secreted proteins. Yeast clones expressing bacterial-binding proteins are isolated by magnetic separation using streptavidin microbeads and are identified by next-generation sequencing of their specific barcode sequences (Fig 1A). Because the expression of *B*. *burgdorferi* virulence factors can vary with temperature, we performed BASEHIT with cultures grown at either 33°C or 37°C. Out of the more than 1000 proteins present in the library, only human peptidoglycan recognition protein 1 (PGLYRP1) exceeded a stringent significance threshold at both temperatures (Fig 1B). We expanded our screen to encompass a wider range of *Borrelia* species and strains, again grown at both 33°C and 37°C, including isolates from diverse geographic origins (S1 Table). We observed that bacteria from the genus *Borrelia* showed significantly greater binding to PGLYRP1 as compared to hundreds of phylogenetically diverse non-*Borrelia* species (Fig 1C). PGLYRP1 was also found to bind to a small number of Gram-positive strains with scores above 3 only, but overall PGLYRP1 binding to *Borrelia* was stronger as compared to most Gram-positive bacteria (S1 File). *B*. *burgdorferi* N40 also exhibited additional predicted interactions with select human extracellular and secreted proteins (S2 Table).

### PGLYRP1 binds and kills *B*. *burgdorferi*

**Fig 2:**
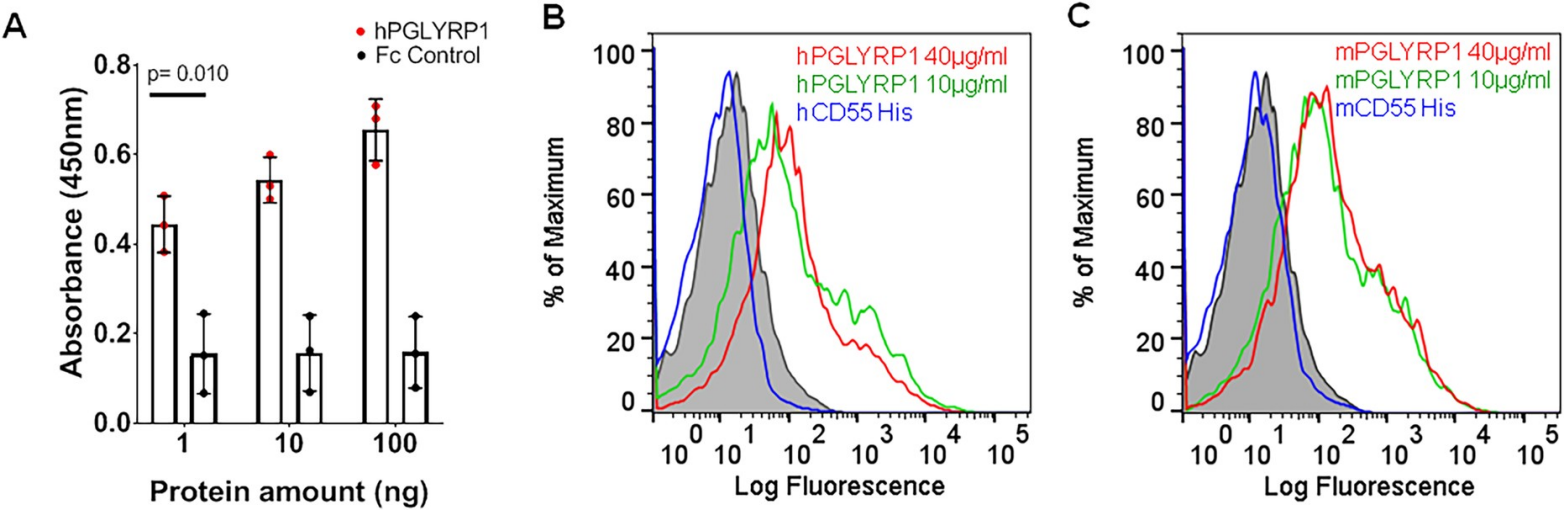
Binding of PGLYRP1 with *B*. *burgdorferi*. (A) ELISA results show the interaction of human PGLYRP1 with lysate of *B*. *burgdorferi*. The lysate was immobilized on microtiter wells and probed with increasing concentrations of either recombinant human PGLYRP1-Fc protein (1–100 ng, red) or Fc control protein (gray). The values plotted represent the mean ± SEM of three replicates from a single experiment. p-value is displayed in the graph and determined using a student t-test. (B and C) Binding of human (B) and mouse (C) PGLYRP1 to *B*. *burgdorferi*. The culture was grown to a density of 10^6^ CFU/mL and incubated with varying concentrations of recombinant PGLYRP1-His_8_ (10 μg/mL in green, 40 μg/ml in red). *B*. *burgdorferi* bound to recombinant PGLYRP1-His_8_ was measured using a secondary AF488-His_6_ monoclonal antibody by flow cytometry. Overlay histograms show protein binding to *B*. *burgdorferi* identified by Alexa Fluor 488-His_6_ monoclonal antibody. Binding of recombinant human/murine CD55-His_8_ (40 μg/ml in blue) to *B*. *burgdorferi* was used as control. The background binding of AF488-His_6_ antibody alone with *B*. *burgdorferi* is shown in gray shaded region. Results from one independent experiment are shown here. For (B) and (C), the Y-axis represents relative cell counts calculated as a percentage of the maximum events (*Borrelia*).

To determine whether PGLYRP1 directly binds to *B*. *burgdorferi* N40, a prototypic isolate commonly used in many laboratories, interaction studies were carried out using sandwich ELISA. We observed a robust, dose-dependent interaction between recombinant Fc-tagged human PGLYRP1 (0–100 ng) and whole cell *B*. *burgdorferi* lysate (Fig 2A). A weak non-specific binding was observed with Fc domain alone, which did not increase with concentration.

To further validate the potential interaction between PGLYRP1 and *B*. *burgdorferi*, we performed flow cytometry based binding assays. Since PGLYRP1 is conserved in humans and mice with 67% identity (S1 Fig), we tested whether mouse PGLYRP1 also recognizes *B*. *burgdorferi*. Both murine and human PGLYRP1 bound to a majority of *B*. *burgdorferi* cells at two different concentrations (10 and 40 μg/ml), while secondary antibody alone showed weak reactivity to spirochetes. As a control, we used recombinant CD55-His_8_ (40 μg/ml), a host immune protein that was predicted by the yeast display screen to be unable to bind *B*. *burgdorferi* (Fig 1). As shown in Fig 2B and 2C, purified murine or human CD55-His_8_ did not recognize *B*. *burgdorferi*. We also observed concentration-dependent binding of human and mouse PGLYRP1 to *B*. *burgdorferi* (S2 Fig), while secondary antibody alone showed only weak binding. Collectively, these results confirmed the interaction between *B*. *burgdorferi* and both human and murine PGLYRP1.

PGLYRP1 is known to be a bactericidal protein expressed by the myeloid cells, primarily neutrophils [19,20]. *B*. *burgdorferi* was incubated with purified human PGLYRP1 for 48 hours in microaerophilic conditions at 33°C. The borreliacidal activity of PGLYRP1 was assessed by BacTiter-Glo Microbial Cell Viability Assay, which has been performed routinely for live *Borrelia* estimation [21–25]. We found that the purified human PGLYRP1 shows borreliacidal activity in a concentration-dependent manner, as shown in S3 Fig. These results suggest that human PGLYRP1 can bind and kill *Borrelia in vitro*.

### Neutrophil mediated phagocytosis and killing of *Borrelia*

Neutrophils kill bacteria in a variety of ways which include the release of granules that contain many microbicidal enzymes [26]. We compared the neutrophils isolated from mouse bone marrow of BALB/c wild type to BALB/c mice that lack an intact *pglyrp1* gene (WT and PGLYRP1^-/-^). Neutrophils were incubated with fluorescent-dye (eFluor 670)-labeled *B*. *burgdorferi* at different ratios. The percent of neutrophils that phagocytosed fluorescent *Borrelia* were analyzed by flow cytometry. Our data shows that PGLYRP1 deficiency does not affect the ability of neutrophils to phagocytose *B*. *burgdorferi* (S4 Fig).

Further, the neutrophil mediated *Borrelia* killing assay showed that there was no significant difference in the borreliacidal activity of neutrophils derived from BALB/c WT and PGLYRP1 knockout mice (S5 Fig). Based on our data, it is apparent that the absence of PGLYRP1 does not affect the ability of neutrophils to kill *Borrelia in vitro*.

### PGLYRP1 binds to *B*. *burgdorferi* peptidoglycan

**Fig 3:**
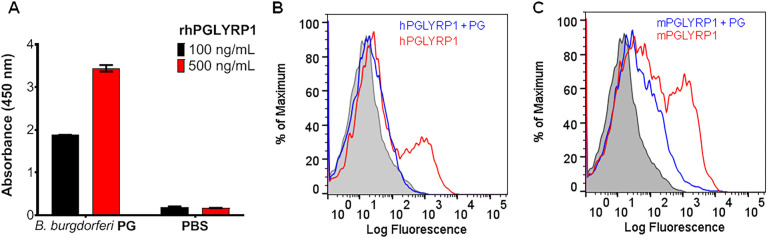
PGLYRP1 shows competitive binding to purified peptidoglycan and *B*. *burgdorferi*. (A) ELISA shows the binding of recombinant human PGLYRP1 at two concentrations, 100 and 500 ng/ml to peptidoglycan (PG) sacculi isolated from *B*. *burgdorferi* (Bb) as compared to a PBS negative control. The values plotted represent the mean ± SEM of two technical replicates from a single experiment. (B and C) Flow cytometry-based experiment showing binding of human (B) and mouse (C) PGLYRP1 to *B*. *burgdorferi*, after pre-incubating the protein in the absence (red) and presence (blue) of *B*. *burgdorferi* PG. Recombinant PGLYRP1-His_6_ (1 μg/mL) was pre-incubated with *Borrelia* PG (10 μg/mL) and then added to live *B*. *burgdorferi* overlay histograms show PGLYRP1 binding to *B*. *burgdorferi* identified by Alexa Fluor 488, His_6_ monoclonal antibody. Results from one independent experiment are shown here. For (B) and (C), the Y-axis represents relative cell counts calculated as a percentage of the maximum events (*Borrelia*).

PGLYRP1 has been shown to have a ligand-binding groove that is specific for peptidoglycan [20]. We, therefore, confirmed the binding of PGLYRP1 with *B*. *burgdorferi* PG. As shown in Fig 3A, a dose-dependent interaction was observed by ELISA between purified PG and hPGLYRP1. Furthermore, flow cytometry-based binding assays revealed that the binding between *B*. *burgdorferi* and human/murine PGLYRP1-His_8_ was reduced with the addition of *B*. *burgdorferi* PG, indicating the specificity of this interaction (Fig 3B and 3C).

### PGLYRP1 influences murine Lyme borreliosis

**Fig 4:**
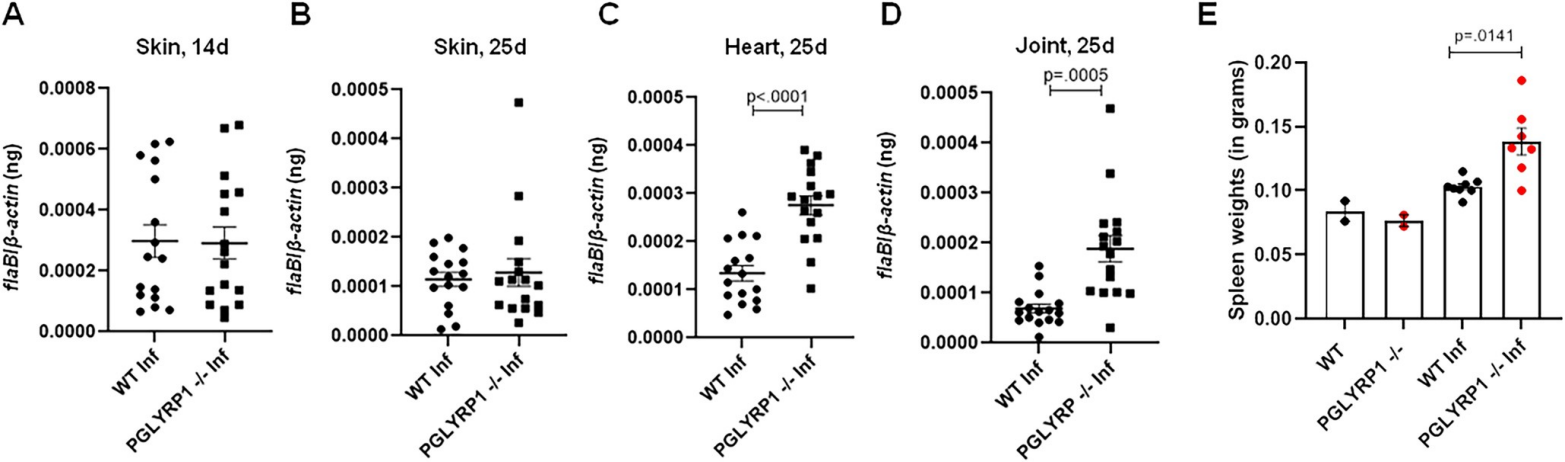
Comparison of *Borrelia* burden in WT BALB/c and PGLYRP1^-/-^ knockout mice. Wild-type BALB/c and PGLYRP1^-/-^ mice (at least n = 7 in each group) were infected with 1x10^6^ spirochetes by subcutaneous injection. (A and B) Skin *B*. *burgdorferi* burden was assessed by ear punch biopsies at 14 d (A) and 25 d (B) post infection, by qPCR for *Borrelia* specific gene (*flaB*) normalized to mouse *β-actin*. (C and D) *B*. *burgdorferi* burden was assessed in hearts (C) and joints (D) at day 25, by qPCR as above. Results from two independent experiments are shown. Each data point represents the value of an individual animal. (E) The extent of splenomegaly was expressed as spleen weights in WT and PGLYRP1^-/-^ mice infected with *B*. *burgdorferi* at day 25. Results from one independent experiment are shown here. Each data point represents the value of an individual animal. The bars represent mean ± SEM and p-values are calculated by student t-test.

To assess the role of PGLYRP1 in the pathogenesis of Lyme borreliosis, we compared the outcomes of *B*. *burgdorferi* infection of BALB/c WT and PGLYRP1^-/-^ mice. BALB/c mice can readily be infected with spirochetes, and while they do not develop severe tenosynovitis like C3H mice, they are useful for the study of murine *B*. *burgdorferi* infection [27]. The BALB/c WT and PGLYRP1^-/-^ mice were infected with 1x10^6^ spirochetes injected subcutaneously. Spirochete burdens in the heart and joint tissues were assessed at 25 days post infection (dpi), and skin burdens were assessed by ear punch biopsies at 14 and 25 dpi. There was no difference between the WT and PGLYRP1^-/-^ mice in the skin burden at either 14 or 25 dpi (Fig 4A and 4B). However, the PGLYRP1^-/-^ mice had a significantly higher spirochete burden in the hearts and joints at 25 dpi (Fig 4C and 4D), suggesting a role for PGLYRP1 in controlling *B*. *burgdorferi* dissemination during the systemic phase of infection. Furthermore, *B*. *burgdorferi* infection induced modest splenomegaly in PGLYRP1^-/-^ mice compared to WT animals, which may represent activation and expansion of splenic immune cells due to higher *B*. *burgdorferi* burden (Fig 4E).

**Fig 5:**
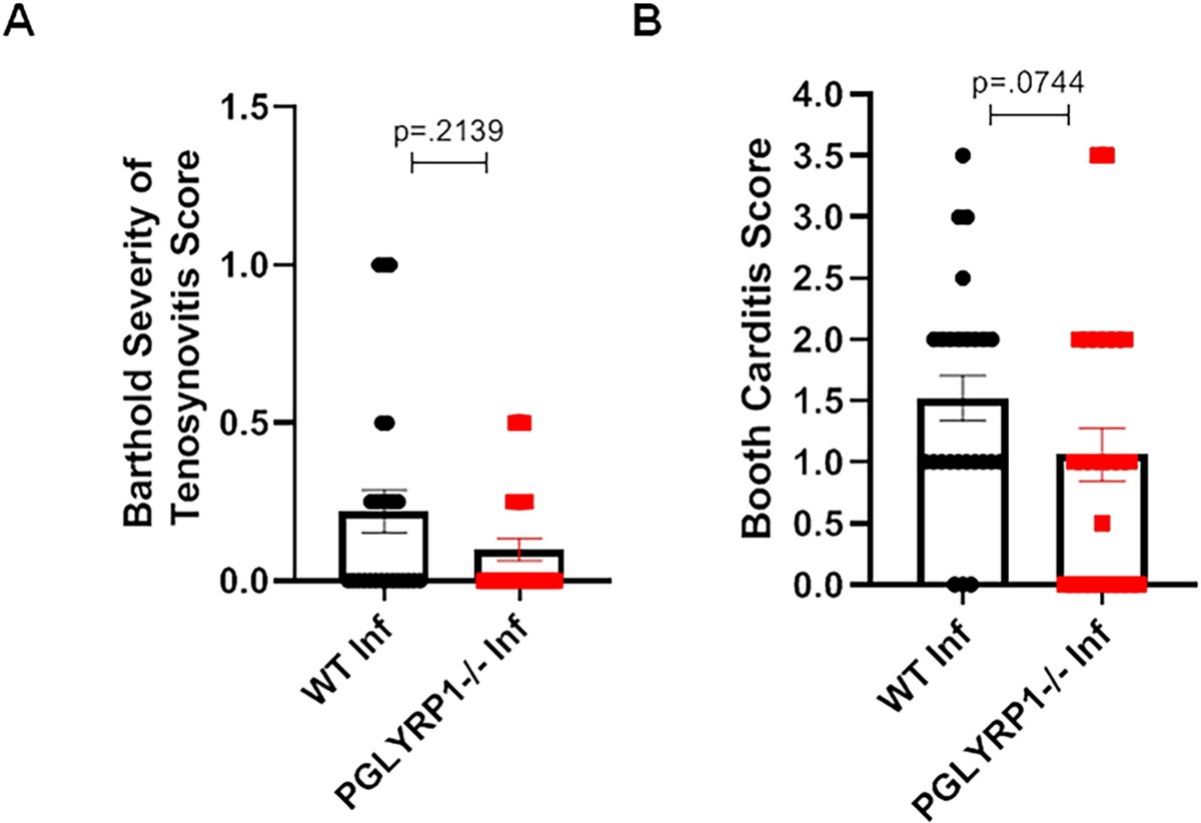
Comparison of tenosynovitis and carditis severity after infection between WT BALB/c and PGLYRP1^-/-^ knockout mice. (A) Histopathology scores from tibiotarsi for individual mice in infected wild-type mice (WT Inf) and infected PGLYRP1^-/-^ mice (PGLYRP1^-/-^ Inf) at 25 days post infection. Tibiotarsi were scored by blinded examination for tenosynovitis on a scale of 0 (negative) to 3 (severe). (B) The severity of cardiac inflammation in the heart of infected WT and PGLYRP1^-/-^ mice 25 d post infection. Hearts were scored in a blinded fashion for carditis on a scale of 0 (negative) to 5 (severe). Similar to tenosynovitis scores, PGLYRP1^-/-^ infected mice demonstrated no significant difference in carditis scores compared to WT mice. Results from at least two independent experiments (at least n = 7 in each group) are pooled and shown here. The bars represent mean ± SEM and p-values were calculated by Mann-Whitney U-test.

Histopathological evaluation was performed to examine the development of acute tenosynovitis in the mice. The ankle (tibiotarsal) joints from WT and PGLYRP1^-/-^ mice (N = 3 uninfected controls per genotype and 25 infected mice per genotype) were examined and assessed for the presence and severity of inflammation. Of the 6 uninfected control mice tibiotarsal joints, only 1 joint (WT tibiotarsal) had trace inflammation (score of 0.5). However, in the 50 tibiotarsal joints examined at 25 dpi, 44% of the tibiotarsal joints from WT and 28% tibiotarsal joints from PGLYRP1^-/-^ mice developed an inflammatory response. Since BALB/c mice do not develop the frequency and severity of tenosynovitis found in C3H mice, we did not find any differences in tenosynovitis severity of tibiotarsi (Fig 5A). These results suggest that, while the lack of *pglyrp1* led to higher *B*. *burgdorferi* burden in animals, joint inflammation was not significantly different.

The hearts from the 6 uninfected control and 50 infected mice 25 dpi described above were examined for the presence and severity of inflammation [28,29]. None of the uninfected control mice were positive for carditis. However, in 50 mice examined at 25 dpi, 84% of the WT and 60% of PGLYRP1^-/-^ infected mice demonstrated inflammation in the heart. There were no significant differences in cardiac severity scores between the groups (Fig 5B). Taken together with the tenosynovitis scores, these data demonstrate that lack of *pglryp1* does not significantly impact the severity of inflammation of hearts and joints in BALB/c mice infected with *B*. *burgdorferi*.

**Fig 6:**
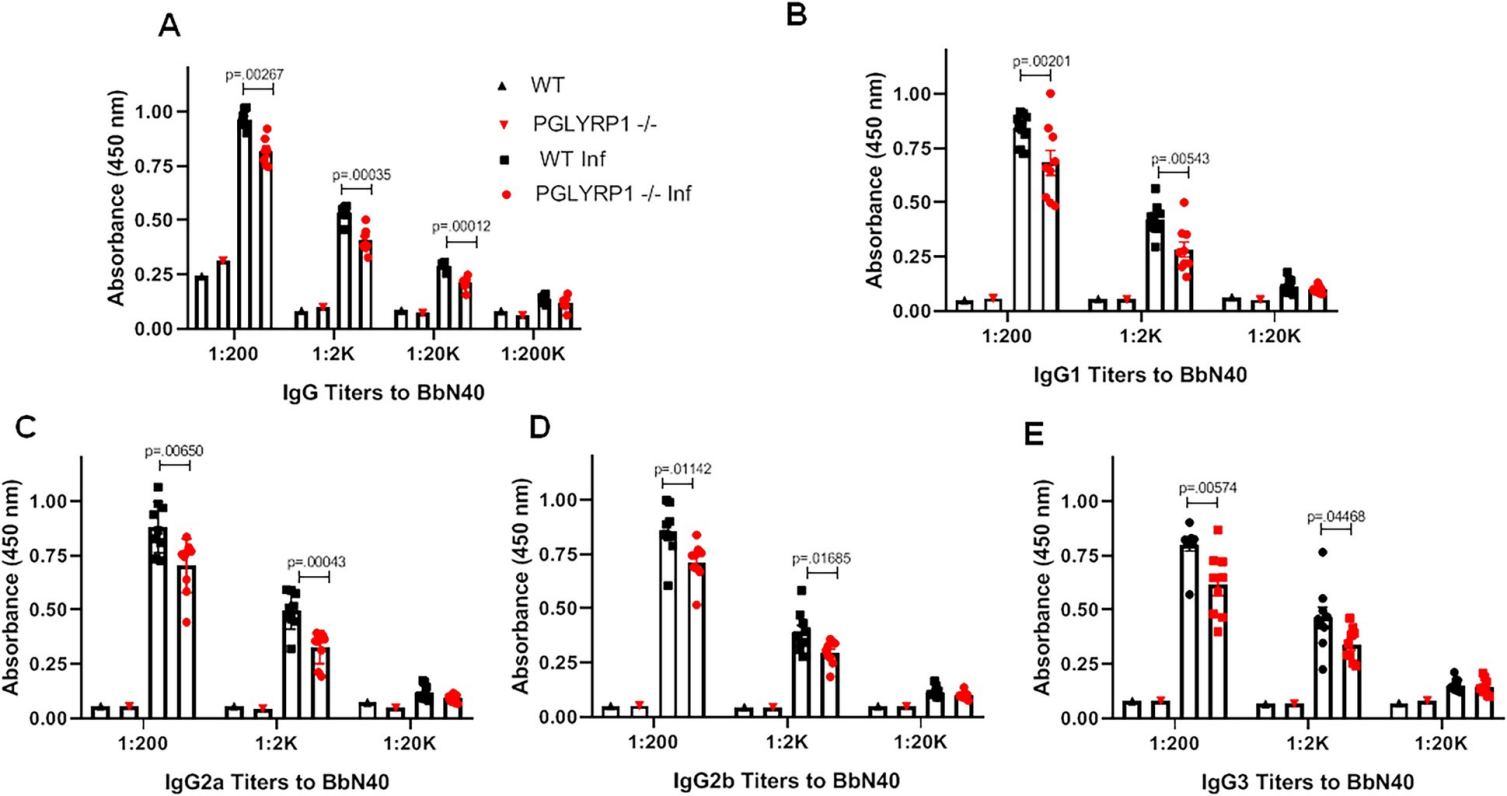
Difference in antibody level after *B*. *burgdorferi* infection in mice. Antibody levels in uninfected wild type BALB/c (WT) and PGLYRP1^-/-^ mice were compared with those in the infected ones (at least n = 7 in each group). Representative results from one independent experiment are shown. (A) Whole cell lysate of *B*. *burgdorferi* was coated on microtiter plate and serum from either uninfected WT, infected WT, uninfected PGLYRP1^-/-^ or infected PGLYRP1^-/-^ mice was used at varying dilutions. The binding was measured by secondary goat anti-mouse IgG HRP-conjugated antibody. Statistically significant increase in binding was observed at IgG titers 1:200, 1:2000, 1:20000 in infected WT compared to infected knockout mice. (B-E) Levels of different IgG isotypes (IgG1, B; IgG2a, C; IgG2b, D; IgG3, E) were measured against *B*. *burgdorferi* lysate, using mouse serum at varying dilutions. The binding was measured by secondary goat anti-mouse IgG1, IgG2a, IgG2b or IgG3 HRP-conjugated antibody. Statistically significant increase was observed in IgG1, IgG2a, IgG3, and IgG2b at 1:200 and 1:2000 dilution in infected WT compared to infected PGLYRP1^-/-^ mice. Representative results from one independent experiment are shown. Each data point represents an individual animal in the corresponding group. The bars represent mean ± SEM and p values determined using student t test.

To determine the effect of PGLYRP1 deletion on the *B*. *burgdorferi* specific antibodies, we measured antibody responses to spirochete antigens via sandwich ELISA. There was a significant reduction in *B*. *burgdorferi*-specific IgG in the sera obtained from PGLYRP1^-/-^ mice compared to WT BALB/c mice, collected at 25 dpi (Fig 6A), while no differences were observed in *B*. *burgdorferi*-specific IgM levels at 25 dpi (S6 Fig). To determine the role of PGLYRP1 on class switching and concomitantly antibody effector functions, we assessed *B*. *burgdorferi* specific IgG1, IgG2a, IgG2b and IgG3 antibodies levels by ELISA. Similar to the overall IgG responses, the levels of *B*. *burgdorferi*-specific IgG1, IgG2a, IgG2b and IgG3 were significantly lower in PGLYRP1^-/-^ infected mice than in WT BALB/c after 25 dpi (Fig 6B–6E).

**Fig 7:**
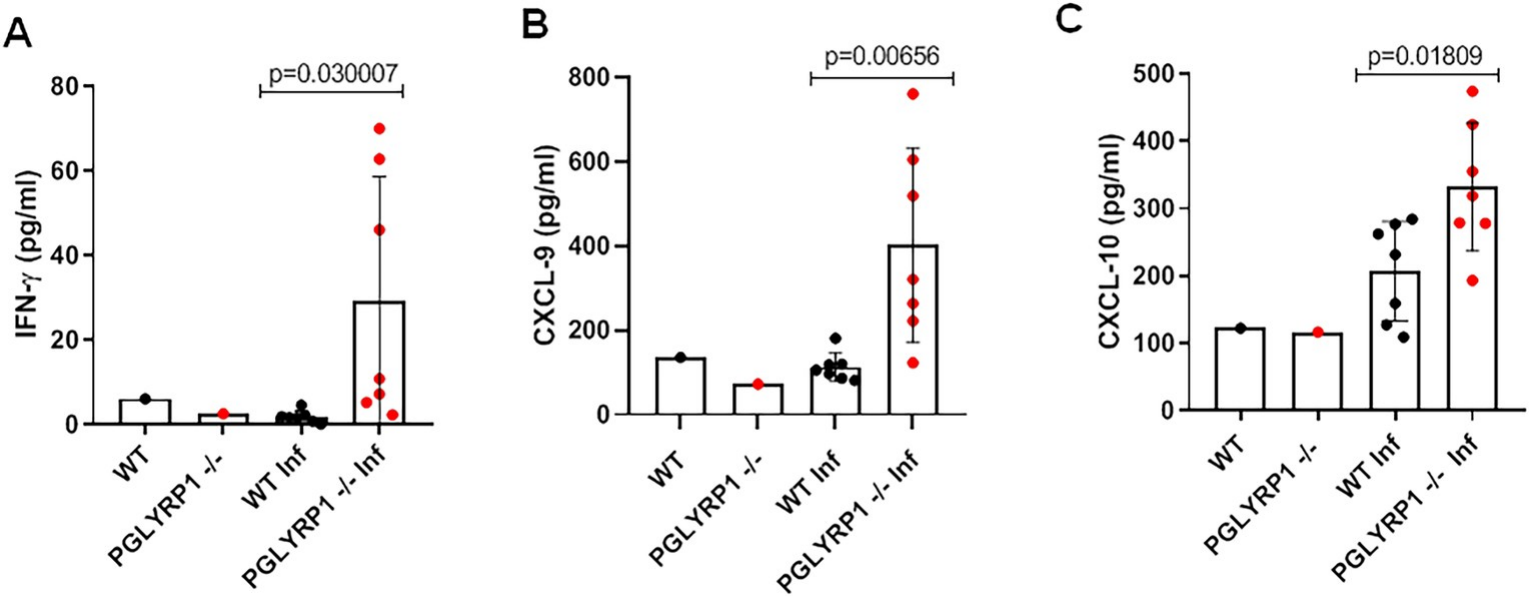
Differential cytokine profile after infection with *B*. *burgdorferi* in mice. Serum cytokine profile was assessed in both wild-type and PGLYRP1^-/-^ mice 25 d post-infection ((at least n = 7 in each group) using a mouse cytokine/chemokine 31-plex (MD-31) array. An increase in pro-inflammatory cytokines IFN-γ (A) and related cytokines- CXCL-9 (B) and CXCL-10 (C) was observed in infected PGLYRP1^-/-^ mice as compared to parent BALB/c mice. Representative results from one independent experiment are shown. Each data point represents an individual animal in the corresponding group. The bars represent mean ± SEM and p-values determined using student t test.

Furthermore, serum cytokine profiles were assessed in both WT and PGLYRP1^-/-^ mice 25 dpi, using a mouse cytokine/chemokine array panel. Sera from uninfected WT and PGLYRP1^-/-^ mice were also probed as baseline controls. Increase in pro-inflammatory cytokines IFN-γ (Fig 7A), and related cytokines CXCL-9 (Fig 7B), CXCL-10 (Fig 7C) was observed in infected PGLYRP1^-/-^ mice as compared to parent WT BALB/c mice. The PGLYRP1^-/-^ mice did not show significant differences in other cytokine levels, including TNF-α, IL-10, IL-4, IL-5, IL-10, IL-17, CXCL-1, MCP-1, MIP-1B, and MIP-2 levels compared to infected WT BALB/c mice (S7 Fig). These results show that the *pglyrp1* absence in the mice increased the Th1 cytokine response, while impairing murine antibody response to *B*. *burgdorferi*.

## Discussion

The pathogenesis of Lyme borreliosis is multifactorial and is associated with the virulence of the pathogen and the host response to *B*. *burgdorferi*. This complex interplay can influence the outcome of the infection and result in varying disease phenotypes [6]. Unbiased combinatorial approaches, such as BASEHIT, present an unparalleled opportunity to identify key host factors that interact with *B*. *burgdorferi*. In this study, we probed 53 different *Borrelia* isolates against 1,031 extracellular human proteins. PGLYRP1 was identified as the most prominent candidate that interacts with multiple *B*. *burgdorferi* isolates grown at 33°C and 37°C.

It has been reported that there are temperature-related changes in *Borrelia* gene expression, which has been shown by different groups [30–32]; Several antigenic lipoproteins present on the *Borrelia* surface have been shown to be induced at 37°C, such as OspC, BbK2.10, ElpA1, Erp proteins and P21 [30–34]. In BASEHIT, we found that the PGLYRP1 interaction with *Borrelia* enhanced marginally at 37°C compared to 33°C. This suggests that temperature-dependent changes in virulence-related gene expression in *Borrelia* may enhance PGLYRP1 binding and may allow us to differentiate between interactions happening at different stages of the spirochete life cycle.

PGLYRP1 is an innate recognition protein that binds to PG. Among the many spirochete components that contribute to pathogenesis, it has recently been shown that *B*. *burgdorferi* sheds PG during growth, and this activity may be associated with inflammation during infection [11]. Using BASEHIT, we found that PGLYRP1 binds more efficiently to *B*. *burgdorferi* spp., as compared to the other bacteria tested. This may be due to the distinct morphology of *B*. *burgdorferi* or the unusual chemical composition of PG [11,35].

In line with this interpretation, we demonstrated direct binding of *B*. *burgdorferi* to PGLYRP1, which could be reduced by supplementation with excess PG. This finding intrigued us since *B*. *burgdorferi* is a diderm bacteria with an outer membrane and a layer of peptidoglycan in the periplasmic space [36]. Peptidoglycan recognition proteins (PGRPs) have been shown to directly bind to peptidoglycan in the bacterial cell wall in Gram-positive bacteria [37]. In our screen, PGLYRP1 did recognize a small number of Gram-positive bacteria. However, it is unclear why all of the Gram-positive bacteria screened were not recognized. There may also be variation in peptidoglycan and peptidoglycan-associated proteins across the Gram-positive bacteria used in this screen.

**Fig 8:**
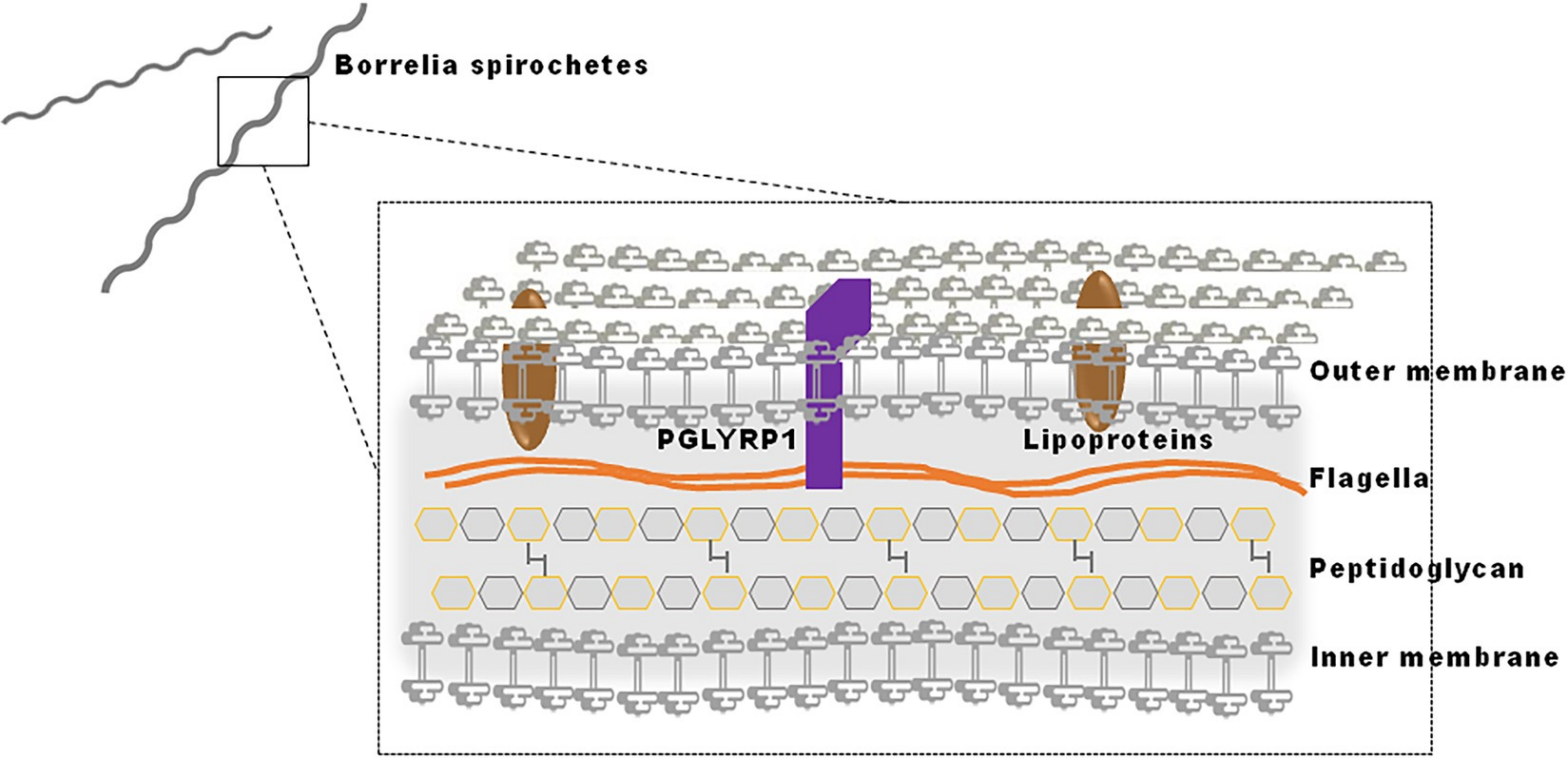
Schematic showing *Borrelia* membrane architecture and PGLYRP1 binding. *B*. *burgdorferi* is a diderm bacteria where the outer membrane surrounds the peptidoglycan layer and protects it from the external environment. The peptidoglycan meshwork, in turn, surrounds the cytoplasm. Our results suggest PGLYRP1 binds to *Borrelia* peptidoglycan.

We think PGLYRP1 can bind to exposed peptidoglycan present on dividing *Borrelia* as well as to damaged spirochete. Spirochetes also have unique peptidoglycan which is continuously shedded and some of the shedded peptidoglycan may be loosely associated with other antigens (Fig 8) [11]. The stronger binding of PGLYRP1 to *Borrelia* could also be due to the unique ornithine within borrelial PG [11]. BASEHIT screen indicates that PGLYRP1 may have a binding preference for unusually modified peptidoglycan in *Borrelia*. It is also possible that PGLYRP1 may differentially recognize cell-surface peptidoglycan (due to cross-linking with associated molecules in the cell wall, etc), compared to free/soluble peptidoglycan, which is why PGLYRP1 only interacted with a small number of Gram-positive bacterial strains while failed to bind many Gram-positive strains tested. For Gram-negative bacteria, PGRPs have been shown to bind uniformly with the outer membrane, which covers the thin layer of peptidoglycan and is composed of lipopolysaccharide (LPS) [38]. In addition to PG, PGRPs bind LPS and other components of the outer membrane using additional binding sites outside of the peptidoglycan-binding groove [37,39–44]. The ability of PGLYRP1 to bind *B*. *burgdorferi* may be completely mediated by PG or involve additional lipoproteins on the outer membrane, given the structural similarity to Gram-negative bacteria [45]. Variations in the surface lipoproteome may account for differences in efficiency scores for PGLYRP1 interaction with the multiple *Borrelia* isolates in BASEHIT. These data indicate that the PGLYRP1 may also bind to additional ligands present on the *Borrelia* surface.

PGLYRP1 is involved in antimicrobial response and has been shown to kill bacteria by inducing oxidative, thiol, and metal stress [46]. In Gram-positive bacteria, PGRPs have direct interaction with PG, which allows for bacterial killing [38,41]. PGRPs are able to induce a stress response in Gram-negative organisms by binding to the outer membrane and activating a two-component system that involves a transmembrane sensor and cytoplasmic regulator, leading to bacterial death [44,47]. Our experiments show that PGLYRP1 had direct bactericidal activity against *Borrelia*. The role of PGLYRP1 in *B*. *burgdorferi* pathogenesis and dissemination is further corroborated by higher load of *B*. *burgdorferi* in hearts and joints of mice lacking PGLYRP1. The enhanced spirochete load in these tissues further substantiates our *in vitro* data that PGLYRP1 can directly kill *Borrelia*. The alteration in antibody responses against *B*. *burgdorferi*, observed *in PGLYRP1*^-/-^ mice also suggests a role for this protein in adaptive immune responses. This may also be partially responsible for higher heart and joint burden, as antibody responses help combat *B*. *burgdorferi* infection [48,49].

*B*. *burgdorferi* exhibits a tropism for heart and joint tissues during murine infection, which are the main sites of inflammation. In many cases, an increase in the *B*. *burgdorferi* burden in mice is associated with increased severity of disease [50,51]. In our study, PGLYRP1 deficiency in BALB/c mice did not result in differences in carditis and tenosynovitis severity, despite a higher pathogen load. This finding is consistent with some studies where disease severity and spirochete numbers do not necessarily correlate [51,52]. Further studies in different strains of inbred mice including animals that develop both severe and mild tenosynovitis, as well as experiments involving different doses and isolates of *B*. *burgdorferi* will be needed to fully elucidate the influence of PGLRYP1 on *Borrelia* pathogenesis.

PGLYRP1 has been shown to modulate inflammation in multiple murine models for experimental colitis, asthma, and contact dermatitis [19,53,54]. In addition, PGLYRP1 can complex with bacterial PG as a ligand for TREM-1 (Trigger receptor expressed on myeloid cells), a pro-inflammatory receptor implicated in innate immune activation [55]. In our study, PGLYRP1^-/-^ mice exhibited a differential cytokine profile with notable increases in serum IFN-γ, CXCL-9, and CXCL-10 levels, favoring a Th1 immune response. The downstream effects of these elevated pro-inflammatory markers remain unclear, as it may be a consequence of higher pathogen burden or directly related to the absence of PGLYRP1. These studies suggest an immunomodulatory role of PGLYRP1; however, further work is warranted to understand these mechanisms in Lyme disease pathogenesis.

In conclusion, we identified novel human immune proteins that bind *B*. *burgdorferi* through an unbiased systems proteomics screen (BASEHIT). Our results show that BASEHIT may be very selective and only identified a small number of immunogenic proteins that could potentially interact with *Borrelia*. However, it is possible that *Borrelia* adhesins may not be accessible enough to be detected or their expression level is too low for the *Borrelia* isolates used in the assay.

To our knowledge, this novel method is the only approach to screen a large number of pathogens and identity interacting partners from a library of more than 1000 human secretory proteins in a simple and efficient manner and will provide a new benchmark in identifying host-pathogen interactions against *Borrelia*. In particular, we uncovered a novel interaction between PGLYRP1 and *B*. *burgdorferi*. We confirmed this interaction using orthogonal methods and identified a role for PGLYRP1 in controlling *B*. *burgdorferi* burden during murine infection. This revealed a new aspect of *B*. *burgdorferi*- host interactions, where PGLYRP1 binds to *Borrelia* cells and kills the rapidly dividing spirochete. *B*. *burgdorferi*, in turn, may be able to subvert this attack by shedding muropeptides that neutralize PGLYRP1 during dissemination to other tissues. PGLYRP1 not only controls *Borrelia* growth through direct interactions, but also indirectly by enhancing effective antibody responses. The role of PGLYRP1 in the humoral response could be due to its involvement in inflammatory signaling and maintaining a balance between Th1/Th2 responses, which may also affect pathogenesis. Further applications of BASEHIT may enable the identification of additional host factors involved in pathogenic processes caused by *Borrelia* and other bacterial species.

## Materials and methods

### Ethics statement

All experiments performed in this study were conducted in accordance with the Guide for the Care and Use of Laboratory Animals. Protocols were approved by the Institutional Animal Care and Use Committee at Yale University (Protocol Permit Number: 07941). All efforts were made to reduce animal suffering during this study.

### *Borrelia* culture

For screening, 53 isolates of several *Borrelia* species were grown at 33°C and 37°C in Barbour-Stoenner-Kelly H (BSK-H) complete medium (Sigma-Aldrich, #B8291) with 6% rabbit serum. The live cell density was ~10^6^−10^7^ cells/mL as determined by dark field microscopy and hemocytometric analysis. The species included various isolates of relapsing fever spirochetes and *B. burgdorferi* sensu lato complex, such as, *B*. *miyamotoi*, *B*. *anserina*, *B*. *crocidurae*, *B*. *duttonii*, *B*. *hispanica*, *B*. *persica*, *B*. *burgdorferi* sensu stricto, *B*. *mayonii*, *B*. *carolinensis*, *B*. *americana*, *B*. *kurtenbachii*, and *B*. *afzelii* (S1 Table). Spirochetes were washed twice with PBS, resuspended in PBS containing 5 μM Sulfo-NHS-LC-Biotin (BioVision, #2326–50), incubated at 37°C for 30 min, washed again with PBS, and glycerol stocks (10% v/v in PBS) were made for future use in yeast display screening assays. A low passage (P<5) clonal isolate of *B*. *burgdorferi N40* grown at 33°C in complete BSK-H medium was used throughout the study.

### Yeast library screening

Details of library construction and selections are described elsewhere (Rosen et al, manuscript in preparation). Briefly, a library of barcoded plasmids containing the extracellular portions of 1031 human proteins was expressed in *Saccharomyces cerevisiae* strain JAR300 and maintained in SDO-Ura (Synthetic drop-out media, US Biological #D9535 prepared with 20 g/L glucose, according to manufacturer's instructions). Expression of surface protein display was induced by culturing the library in media containing 90% galactose and 10% glucose for 24 hours at 30°C. 1x10^7^ Induced yeast cells were harvested in a sterile 96-well v-bottom microtiter plate. Yeast cells were resuspended in 100 μL PBE (PBS + 0.5% w/v BSA + 0.5 mM EDTA) with 10 μL of biotinylated bacteria added and incubated for 1 hour at 4°C with shaking. Yeast cells were washed once with 200 μL PBE, resuspended in 100 μL PBE containing 1 μL of streptavidin microparticles (Spherotech, 0.29 μm, #SVM-025-5H) and incubated for 1 hour at 4°C with shaking. Yeast were washed once with 200 μL PBE and bead bound cells were selected by magnetic separation and subsequently expanded in 1 mL SDO-Ura supplemented with chloramphenicol at 30°C. DNA was extracted from selected yeast cell libraries using Zymoprep-96 Yeast cell Plasmid Miniprep kits or Zymoprep Yeast cell Plasmid Miniprep II kits (Zymo Research) according to standard manufacturer protocols. DNA was amplified with custom primers and sequenced using an Illumina MiSeq and Illumina v2 MiSeq Reagent Kits according to standard manufacturer protocols. Barcode counts were extracted from raw NGS data using Python.

All enrichment calculations were performed using edgeR [56]. The score for each gene is defined as the overall enrichment for that gene (relative to the unselected library) multiplied by the percentage of barcodes associated with the gene that enriched (defined as logFC >0). Since a negative log fold change means a protein was depleted, so for simplicity, all negative values are set to zero.

### ELISA for analysis of *B*. *burgdorferi* interaction with human PGLYRP1

Spirochetes were grown to the density of ~10^7^ cells/mL and harvested at 5000 x g for 15 minutes. Cells were washed twice with PBST (PBS containing 0.05% Tween-20), pelleted and lysed using Bug-buster Protein Extraction Reagent (Novagen, #70921–3), as previously described [57]. Protein concentration in the lysate was measured by absorbance at 280 nm. In a 96-well plate, wells were coated with 100ng of *B*. *burgdorferi* lysate. Samples were blocked with 1% BSA followed by incubation with either recombinant human PGLYRP1 conjugated with an Fc tag or a control protein fused with an Fc tag at varying concentrations (1 ng-100 ng) for 1 hour at 37°C. After washing and incubating with Goat anti-human IgG HRP conjugated secondary antibody (Sigma-Aldrich, #AP309P) (1:5000), KPL Sureblue TMB Microwell Peroxidase substrate, 1-component (Seracare, #5120–0077) was added. The reaction was stopped with 2 M sulfuric acid, and absorbance was read at 450 nm.

### Human PGLYRP1 binding to *B*. *burgdorferi* PG

PG was purified as previously described [10,11,58]. 96-well flat-bottom microplates were coated overnight at room temperature with poly-L-lysine solution (0.1% w/v). Wells were washed twice with PBST and 100 μL of intact PG sacculi (1μg/mL in PBS) isolated from *B*. *burgdorferi* B31 MI was added to duplicate wells, with PBS alone as a negative control. After overnight incubation at 4°C, wells were washed twice with PBST and blocked with PBS containing 3% BSA. After washing, recombinant human PGLYRP1 (R&D Systems, #2590-PGB) was added to each well at a concentration of 500 ng/mL in PBS containing 1% BSA. After incubating for 2 hours at 30°C, wells were washed with PBST and 100 μL 6X-His Tag polyclonal antibody, HRP (Invitrogen, #MA1-135-A488) was added at 1:20,000 dilution. Plates were incubated for 1 hour at room temperature with shaking, followed by washing with PBST and adding 1-Step Ultra TMB-ELISA Substrate Solution (Seracare, #52-00-03). After allowing color development to proceed for 10 minutes, reactions were stopped by addition of 2 M sulfuric acid and the absorbance was measured at 450 nm.

### Borreliacidal assay

The BacTiter Glo microbial cell viability assay provides a method for determining the number of viable microbial cells in culture based on quantitation of the ATP present by measuring luminescence. The luminescent signal is proportional to the amount of ATP present, which is directly proportional to the number of viable cells in culture [21–25]. To test the borreliacidal activity of human PGLYRP1-His_8_ (0–66.7 ng/μl), we incubated the protein with 1x10^5^ spirochetes in microaerophilic conditions at 33°C for 48 h in a final volume of 300 μL, and calculated the percent viable spirochetes using BacTiter-Glo (Promega) as described previously [21–25].

### Flow cytometry based PGLYRP1 and PG binding assay

Low passage *B*. *burgdorferi* were cultured to a density of ~10^6^−10^7^ cells/mL, washed two times with PBS and incubated with either recombinant human PGLYRP1 (with 8X-His tag), recombinant mouse PGLYRP1 (with 6X-His tag) (Sino Biological, #50115-M08H), recombinant human CD55 (with 8X-His tag), or murine CD55 (with 8X-His tag) at room temperature for 1 hour. Varying concentrations of purified *B*. *burgdorferi* PG [11] were used for this assay. PG preparation had been sonicated prior to use and consisted of fragmented sacculi. *Borrelia* PG was pre-incubated with either murine or human PGLYRP1 and was subsequently added to *Borrelia* to test for binding. After a co-incubation period of 1 hour, spirochetes were fixed in 4% PFA, washed three times with PBS and blocked in 1% BSA overnight at 4°C. The spirochetes were probed anti 6X-His monoclonal antibody- conjugated to Alexa Fluor 488 (ThermoFisher, #MA1-21315-488) and run through SA3800 Spectral Analyzer (Sony Biotechnology). The data was analyzed by FlowJo.

### *In vivo* infection of mice

Pathogen-free BALB/c mice wild-type (WT) (Jackson Laboratory) and BALB/c: Pglyrp1^tm1Rdz^/Pglyrp1^tm1Rdz^ (PGLYRP1^-/-^) knockout mice [53,59], 6 to 8 weeks of age were infected with low passage *B*. *burgdorferi* (1x10^6^ spirochetes subcutaneously, 7–9 mice/group). Uninfected WT and PGLYRP1^-/-^ BALB/c mice were used as controls. Ear punches were taken at 14- and 25-days post-infection to compare the *Borrelia* burden in the skin. At 25 days post infection (dpi), heart and joint tissue punches were also collected to quantify the spirochete levels. The spleen weights were measured to assess splenomegaly. The protocol for the use of mice was reviewed and approved by the Yale Animal Care and Use Committee.

### Phagocytosis assay

Bone marrow cells from BALB/c WT and PGLYRP1^-/-^ mice were harvested and neutrophils were separated using density gradient centrifugation [60]. *B*. *burgdorferi* were labeled with eBioscience Cell Proliferation Dye eFluor 670 (ThermoFisher, #65-0840-85) which binds to any cellular protein containing primary amines, and the spirochetes were washed multiple times to remove excess dye. Neutrophils were kept in RPMI1640 culture medium, supplemented with 10% (vol/vol) fetal bovine serum. Neutrophils and spirochetes were mixed in different ratios from 1:1 to 1:30. The suspensions were incubated for 60 minutes at 37°C in the presence of 5% CO_2_ in 96-well U-bottom plate. After incubation, the suspensions were centrifuged at 500x g for 10 min. The pellets were washed twice with PBS, fixed in 2% PFA, and kept at 4°C until further processing. The cells were run through LSRII flow cytometer and analyzed by FlowJo software. Neutrophils were gated based on forward and side scatter, and *Borrelia* phagocytosing cells were identified by fluorescence detected with a 660/20 band pass filter in APC channel. The data is represented as % of neutrophils positive for eFluor 670.

### Neutrophil killing assay

Bone marrow cells from BALB/c WT and PGLYRP1^-/-^ mice were harvested and neutrophils were separated using density gradient centrifugation [60]. Neutrophils were kept in DMEM culture medium, supplemented with 10% (vol/vol) fetal bovine serum. Neutrophils and *B*. *burgdorferi* (1X10^5^) were mixed in the ratio of 1:1 in the absence or the presence of mouse serum (10%). The suspensions were incubated for 60 minutes at 37°C in the presence of 5% CO_2_ in a 96-well flat-bottom plate in 50 μL. After incubation, 250 μL BSK-H media was added to the suspensions and kept at 33°C for 72 hours. The *Borrelia* numbers were counted in Neubauer chamber under the microscope. The experiment was performed three times in triplicates.

### Quantification of *Borrelia* burden

DNA was extracted from heart, joint, and ear punch samples using Qiagen DNeasy Blood & Tissue Kit, Qiagen. Quantitative PCR was performed using iQ-SYBR Green Supermix (Bio-Rad). For quantitative detection of *B*. *burgdorferi* burden within mouse tissue samples, q-PCR was performed with DNA using *flagellin* (*flaB*), a marker gene for *Borrelia* detection. The mouse *β-actin* gene was used to normalize the amount of DNA in each sample. The nucleotide sequences of various primers used in specific PCR applications are indicated in S3 Table.

### Antibody titers against *B*. *burgdorferi*

IgG and IgM titers against *B*. *burgdorferi* were detected in mouse sera by ELISA, as described above. Briefly, wells were coated with lysate of *B*. *burgdorferi*, blocked, and incubated with mouse sera diluted in 1% BSA at varying titers (1:200, 1:2000, 1:20,000, 1:200,000). After washing, Goat anti-mouse IgM HRP conjugated antibody (1:10,000, ThermoFisher #62–6840) or Rabbit anti-mouse IgG HRP conjugated antibody (1:10,000, ThermoFisher, #61–6520) or Goat anti-mouse IgG1/IgG2a/IgG2b/IgG3 HRP (1:10,000, Abcam, ab97240, ab97241, ab97250, ab97260) antibody was added. KPL Sureblue TMB Microwell Peroxidase substrate, 1-component was added, and the reaction was stopped with 2 M sulfuric acid. The absorbance of wells was read at 450 nm.

### Cytokine profile

Serum collected from each group of mice was sent for cytokine analysis by the Mouse Cytokine/Chemokine Array 31-plex (MD-31) performed by Eve Technologies. The cytokines represented by this array are Eotaxin, G-CSF, GM-CSF, IFN-γ, IL-1α, IL-1β, IL-2, IL-3, IL-4, IL-5, IL-6, IL-7, IL-9, IL-10, IL-12 (p40), IL-12 (p70), IL-13, IL-15, IL-17A, IP-10, KC, LIF, LIX, MCP-1, M-CSF, MIG, MIP-1α, MIP-1β, MIP-2, RANTES, TNFα, and VEGF.

### Heart and joint histopathology analysis

Mice were euthanized by CO_2_ asphyxiation and the heart and one rear leg from each mouse were immersion-fixed in 10% neutral buffered formalin (NBF) (hearts) and Bouin’s (Ricca Chemical Corp.) or 10% solution NBF followed by Decal Solution (rear leg). Hearts were bisected and then tissues were processed, embedded, sectioned and stained with hematoxylin and eosin (HE) by routine methods (Comparative Pathology Research Core in the Department of Comparative Medicine, Yale School of Medicine). The tibiotarsi were scored in a blinded fashion for tenosynovitis severity using the 3 point Barthold scoring system: 0 (negative), 1 (minimal), 2 (moderate), to 3 (severe), by an experienced veterinarian (CJB, trained in veterinary pathology), as previously described [29,61]. The hearts were also scored in a blinded fashion for carditis severity on the Booth 5 point scale system of 0 (negative), 1 (minimal), 2 (mild), 3 (moderate), 4 (marked) to 5 (severe), as previously described [29].

### Gene cloning and expression

Human PGLYRP1 (amino acids 22–196) or CD55 (human amino acids 35–353 or mouse amino acids 35–362) was cloned into pEZT-Dlux, a modified pEZT-BM vector. Modifications included insertion of an H7 Leader Sequence followed by an AviTag (Avidity), HRV 3C site, protein C epitope, and an 8X His tag. Modified pEZT-BM vector was a kind gift from Ryan Hibbs (Addgene plasmid #74099). Expi293 cells (ThermoFisher, #A14527) were transfected with the PGLYRP1 or CD55 expression plasmid using ExpiFectamine 293 Transfection Kit (ThermoFisher, # A14524). Protein was purified from clarified media by nickel-nitrilotriacetic acid chromatography and desalted into PBS. For Fc-tagged proteins, human PGLYRP1 (amino acids 22–196) was cloned into pD2610-v1 (ATUM Bio) as an N-terminal fusion to the Fc fragment of human IgG1, with an N297A mutation to eliminate all effector functions, and a 3X (GGGGS) linker sequence. Expi293 cells (Thermo Fisher) were transfected with the PGLYRP1 or control Fc expression plasmid (containing the Fc fragment and linker sequence) using ExpiFectamine 293 Transfection Kit (Thermo Fisher). Protein was purified from the supernatant 96 hours post-transfection using Protein A sepharose (Gold Biotechnology). The column was washed with PBS and eluted using 100 mM glycine pH 3.0. Eluted protein was immediately neutralized with 1 M Tris pH 8.0 and desalted into PBS. Protein purity was verified by SDS-PAGE. Protein concentration was measured by absorbance at 280 nm.

### Statistical analysis

The analysis of all data was performed by Student’s t-test, Mann-Whitney, or ANOVA in Prism 8.0 software (GraphPad Software, Inc., San Diego, CA). A p-value of <0.05 was considered statistically significant.

## Supporting information

S1 Table *Borrelia* species & number of corresponding isolates screened. (DOCX)

S2 Table Top hits from yeast display screen for *B*. *burgdorferi N40* in order of descending enrichment scores. (DOCX)

S3 Table Primers used in this study. (DOCX)

S1 Fig Multiple sequence alignment of human and mouse PGLYRP1. (TIF)

S2 Fig Concentration-dependent binding of human PGLYRP1 to *B*. *burgdorferi*. Spirochetes from *B*. *burgdorferi* culture were incubated with increasing concentrations of (A) recombinant human PGLYRP1-His_8_, and (B) recombinant mouse PGLYRP1-His_6_, at concentrations 0.01 μg/mL, 0.1 μg/mL and 1 μg/ml. Representative results from one independent experiment are shown. Each data point represents an individual animal in the corresponding group. Data is expressed as the percentage of spirochetes that were shown to bind. The Y-axis represents relative cell counts calculated as percentage of the maximum events (*Borrelia*).
(TIF)

S3 Fig PGLYRP1 dependent killing of *B*. *burgdorferi*. Human PGLYRP1- His_8_ (0–66.7 ng/μl) was incubated with *B*. *burgdorferi* (1x10^5^) for 48 hours in 96-well plate, keeping final volume 300 μl. The viability was assessed by BacTiter Glo assay. The graph shows effect of human PGLYRP1 concentration on *Borrelia* (BbN40). Results from one independent experiment performed in triplicates are shown here.
(TIF)

S4 Fig *In vitro* phagocytosis assay using neutrophils from wild type (WT) and PGLYRP1^-/-^ mice. The neutrophils isolated from mouse bone marrow were incubated with eFluor 670 dye-labeled *B*. *burgdorferi* at different ratios for 1 hour. The neutrophils were subsequently washed and analyzed by flow cytometer. (A) Histogram showing neutrophils that phagocytosed *Borrelia*. High and low represent the ratio of 30 and 1 (*B*. *burgdorferi*, 1x10^5^). Y-axis represents relative cell counts calculated as percentage of the maximum events (*Borrelia*). (B) The graph show percent of phagocytic neutrophils plotted against *Borrelia* numbers. Results from three independent experiments are shown. The bars represent mean ± SEM and p-values were determined by Student t-test.
(TIF)

S5 Fig Comparison of *Borrelia* killing using neutrophils from WT and PGLYRP1^-/-^ mice. Neutrophils isolated from mouse bone marrow were incubated with 1x10^5^ *B*. *burgdorferi* for 1 hour in DMEM media in the absence (A) or presence of 10% mouse serum (B). The assay was performed in 96-well flat-bottom plates and volume was 50 μl. After 1 hour, 250 μl of BSK-H medium was added to the wells. The plates were incubated in microaerophilic conditions at 33°C for 72 hours. The *Borrelia* numbers were counted in Neubauer chamber under the dark-field microscope. The experiment was performed in triplicates and results from one independent experiment are shown. The bars represent mean ± SEM and p-values were determined by the Mann-Whitney test.
(TIF)

S6 Fig *B*. *burgdorferi* specific IgM titers in mice. Antibody levels in uninfected wild type BALB/c (WT) and PGLYRP1^-/-^ mice were compared with those in the infected ones (at least n = 7 in each group). Results from two independent experiment are shown. Whole-cell lysate of *B*. *burgdorferi* was coated on a microtiter plate and serum from either uninfected WT, infected WT, uninfected PGLYRP1^-/-^ or infected PGLYRP1^-/-^ mice was used at 1:200 dilution. The binding was measured by the secondary Goat anti-mouse IgM HRP-conjugated antibody. No significant difference in IgM level was observed in infected WT compared to infected PGLYRP1 knockout mice. Each data point represents an individual animal in the corresponding group. The bars represent mean ± SEM and p-values determined by student t-test.
(TIF)

S7 Fig Cytokine response in WT and PGLYRP1^-/-^ mice. The infected PGLYRP1^-/-^ mice also showed overall different levels (in pg/mL) of CXCL-1, MCP-1, MIP-1B, and MIP-2 (A) and TNF-α, IL-10, IL-4, IL-5, IL-10, IL-17 (B) as compared to BALB/c mice infected mice although the profiles were statistically insignificant. Representative results from one independent experiment are shown. Each data point represents an individual animal in the corresponding group. The bars represent mean ± SEM.
(TIF)

S1 File Excel spreadsheet containing the numerical data for Figure panels Fig 1B and Fig 1C in separate sheet tabs; BASEHIT scores for PGLYRP1 and corresponding *Borrelia* isolate (“*Borrelia* PGLYRP1 Scores”); BASEHIT scores for PGLYRP1 for non-*Borrelia* isolates (“Non-*borrelia* PGLYRP1 Scores”; list of *Borrelia* isolates used and corresponding species classification (“*Borrelia* isolates”), source and reference; and list of non-*Borrelia* isolates used with corresponding Gram stain and reference if applicable (“non-*Borrelia* isolates”). (XLSX)

S2 File Excel spreadsheet containing data underlying the figures. (XLSX)
