# Supplementary material for: A human secretome library screen reveals a role for Peptidoglycan Recognition Protein 1 in Lyme borreliosis
Source: PLoS Pathog. 2020 Nov 11;16(11):e1009030. doi: 10.1371/journal.ppat.1009030 (PMC7657531; doi:10.1371/journal.ppat.1009030)
Supplement: S1 Table — (DOCX) [file ppat.1009030.s001.docx]

**S1 Table.** *Borrelia* species & number of corresponding isolates screened.

| **Species** | **Number of Isolates Screened** |
| --- | --- |
| ***Borrelia burgdorferi* sensu lato complex** | **36** |
| *B. afzelii* | 18 |
| *B. americana* | 1 |
| *B. burgdorferi* sensu stricto | 8 |
| *B. carolinensis* | 3 |
| *B. garinii* | 3 |
| *B. mayonii* | 1 |
| *B. kurtenbachii* | 1 |
| *B. valaisiana* | 1 |
|  | |
| **Relapsing fever spirochetes** | **17** |
| *B. anserina* | 1 |
| *B. crocidurae* | 1 |
| *B. duttonii* | 1 |
| *B. hispanica* | 1 |
| *B. miyamotoi* | 12 |
| *B. persica* | 1 |
