# Supplementary material for: A human secretome library screen reveals a role for Peptidoglycan Recognition Protein 1 in Lyme borreliosis
Source: PLoS Pathog. 2020 Nov 11;16(11):e1009030. doi: 10.1371/journal.ppat.1009030 (PMC7657531; doi:10.1371/journal.ppat.1009030)
Supplement: S2 Table — (DOCX) [file ppat.1009030.s002.docx]

**S2 Table.** Top hits from yeast display screen for *B. burgdorferi N40* in order of descending enrichment scores

| **Protein** | **Function with Panther family annotation** | ***B. burgdorferi* 33°C score - average bacterial score** | **p-value relative to average bacteria score** |
| --- | --- | --- | --- |
| PGLYRP1 | peptidoglycan immune receptor activity | 4.029706862 | 2.65251E-05 |
| LAIR2 | inhibitory receptor present on mononuclear leukocytes | 2.49646777 | 0.015449324 |
| LRRTM2 | synapse organization | 1.512091167 | 0.292351992 |
| REG4 | mannose, phospholipase, lectin receptor related pthr22803 | 0.984685246 | 0.246039954 |
| NRP2 | vascular endothelial growth factor-activated receptor activity | 0.924634238 | 0.292351992 |
| TYRO3 | [tyrosine-protein kinase receptor pthr24416](http://www.pantherdb.org/panther/lookupId.jsp?id=PTHR24416) | 0.796971217 | 0.292351992 |
| TLR3 | MyD88-independent toll-like receptor signaling pathway | 0.787420402 | 0.096911657 |
| TMEM130 | [melanocyte protein pmel 17-related pthr11861](http://www.pantherdb.org/panther/lookupId.jsp?id=PTHR11861) | 0.749663357 | 0.099486635 |
| GP1BB | [glycoprotein ib beta pthr22650](http://www.pantherdb.org/panther/lookupId.jsp?id=PTHR22650) | 0.679232994 | 0.292351992 |
| IL32 | pro-inflammatory [cytokine](https://en.wikipedia.org/wiki/Cytokine) that act on [monocytes](https://en.wikipedia.org/wiki/Monocyte) and [macrophages](https://en.wikipedia.org/wiki/Macrophage) to secrete inflammatory cytokines | 0.597363001 | 0.157754405 |
