# Supplementary material for: A human secretome library screen reveals a role for Peptidoglycan Recognition Protein 1 in Lyme borreliosis
Source: PLoS Pathog. 2020 Nov 11;16(11):e1009030. doi: 10.1371/journal.ppat.1009030 (PMC7657531; doi:10.1371/journal.ppat.1009030)
Supplement: S3 Table — (DOCX) [file ppat.1009030.s003.docx]

**S3 Table.** Primers used in this study.

| **Primer** | **Sequence** |
| --- | --- |
| *mactin*_F | AGCGGGAAATCGTGCGTG |
| *mactin*_R | CAGGGTACATGGTGGTGCC |
| *flaB*_F | TTCAATCAGGTAACGGCACA |
| *flaB*_R | GACGCTTGAGACCCTGAAAG |
| *cDNA*_F | CTGTTATTGCTAGCGTTTTAGCA |
| *cDNA*_R | GCCACCAGAAGCGGCCGC |
