# Supplementary figures and images for: A human secretome library screen reveals a role for Peptidoglycan Recognition Protein 1 in Lyme borreliosis
Source: PLoS Pathog. 2020 Nov 11;16(11):e1009030. doi: 10.1371/journal.ppat.1009030 (PMC7657531; doi:10.1371/journal.ppat.1009030)

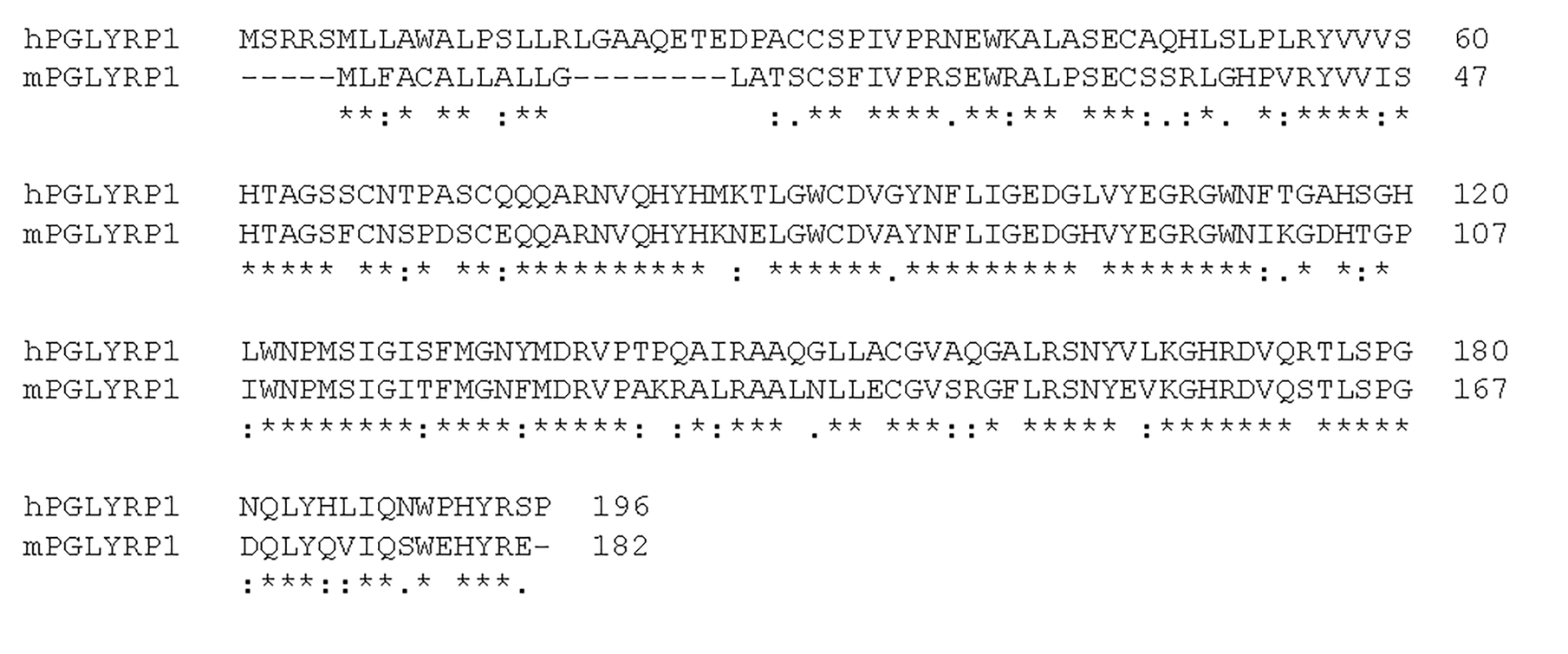

Supplement: S1 Fig — (TIF) [file ppat.1009030.s004.tif]

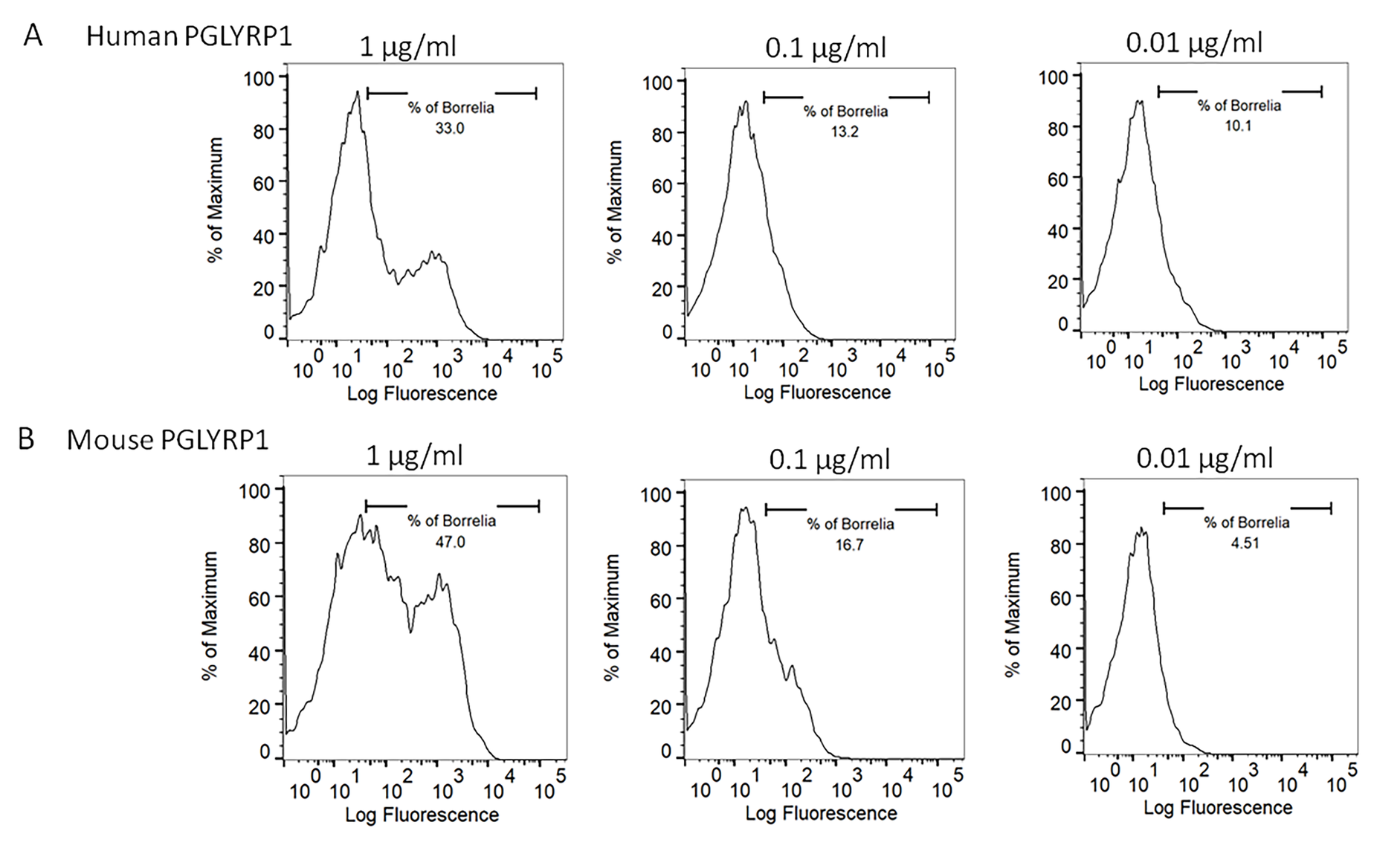

Supplement: S2 Fig — Spirochetes from B. burgdorferi culture were incubated with increasing concentrations of (A) recombinant human PGLYRP1-His8, and (B) recombinant mouse PGLYRP1-His6, at concentrations 0.01 μg/mL, 0.1 μg/mL and 1 μg/ml. Representative results from one independent experiment are shown. Each data point represents an individual animal in the corresponding group. Data is expressed as the percentage of spirochetes that were shown to bind. The Y-axis represents relative cell counts calculated as percentage of the maximum events (Borrelia). (TIF) [file ppat.1009030.s005.tif]

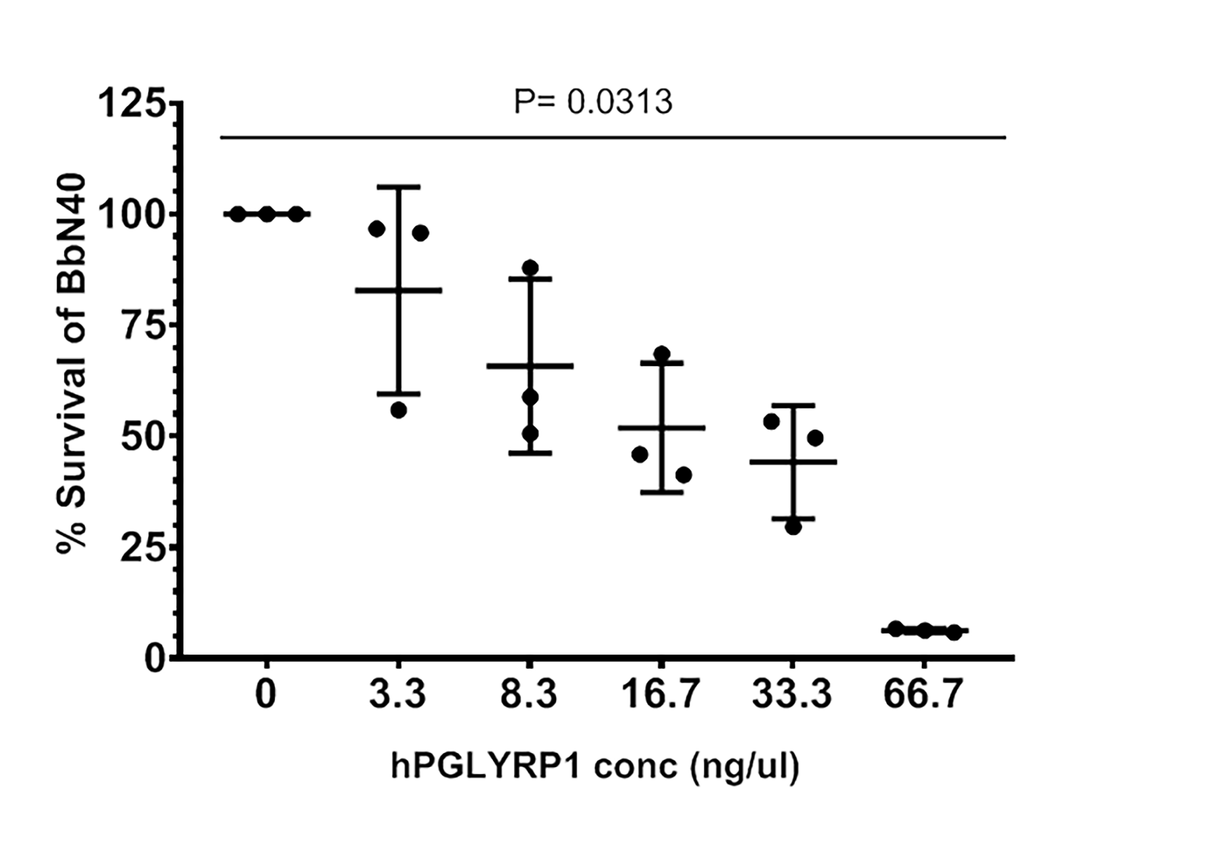

Supplement: S3 Fig — Human PGLYRP1- His8 (0–66.7 ng/μl) was incubated with B. burgdorferi (1x10^5^) for 48 hours in 96-well plate, keeping final volume 300 μl. The viability was assessed by BacTiter Glo assay. The graph shows effect of human PGLYRP1 concentration on Borrelia (BbN40). Results from one independent experiment performed in triplicates are shown here. (TIF) [file ppat.1009030.s006.tif]

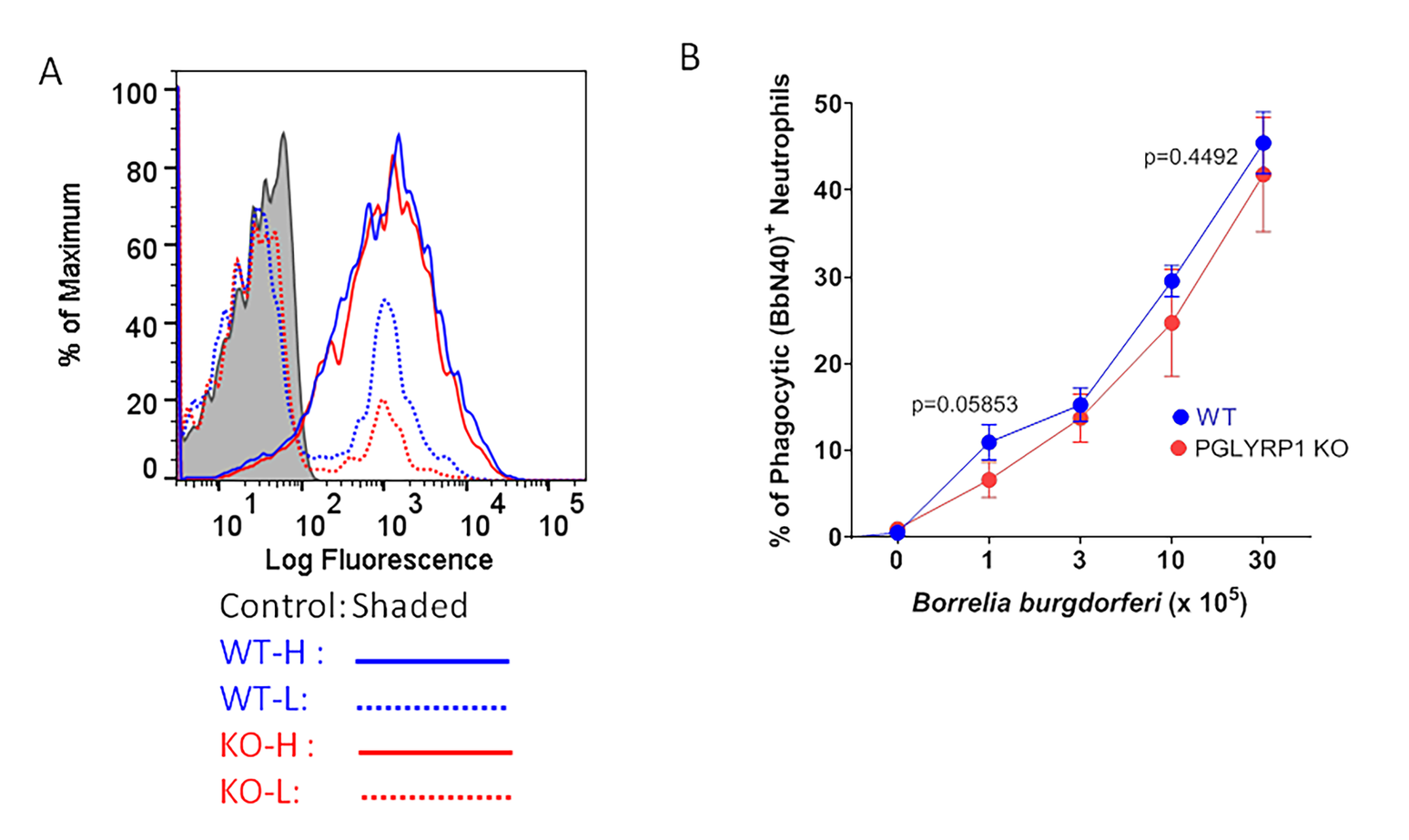

Supplement: S4 Fig — The neutrophils isolated from mouse bone marrow were incubated with eFluor 670 dye-labeled B. burgdorferi at different ratios for 1 hour. The neutrophils were subsequently washed and analyzed by flow cytometer. (A) Histogram showing neutrophils that phagocytosed Borrelia. High and low represent the ratio of 30 and 1 (B. burgdorferi, 1x10^5^). Y-axis represents relative cell counts calculated as percentage of the maximum events (Borrelia). (B) The graph show percent of phagocytic neutrophils plotted against Borrelia numbers. Results from three independent experiments are shown. The bars represent mean ± SEM and p-values were determined by Student t-test. (TIF) [file ppat.1009030.s007.tif]

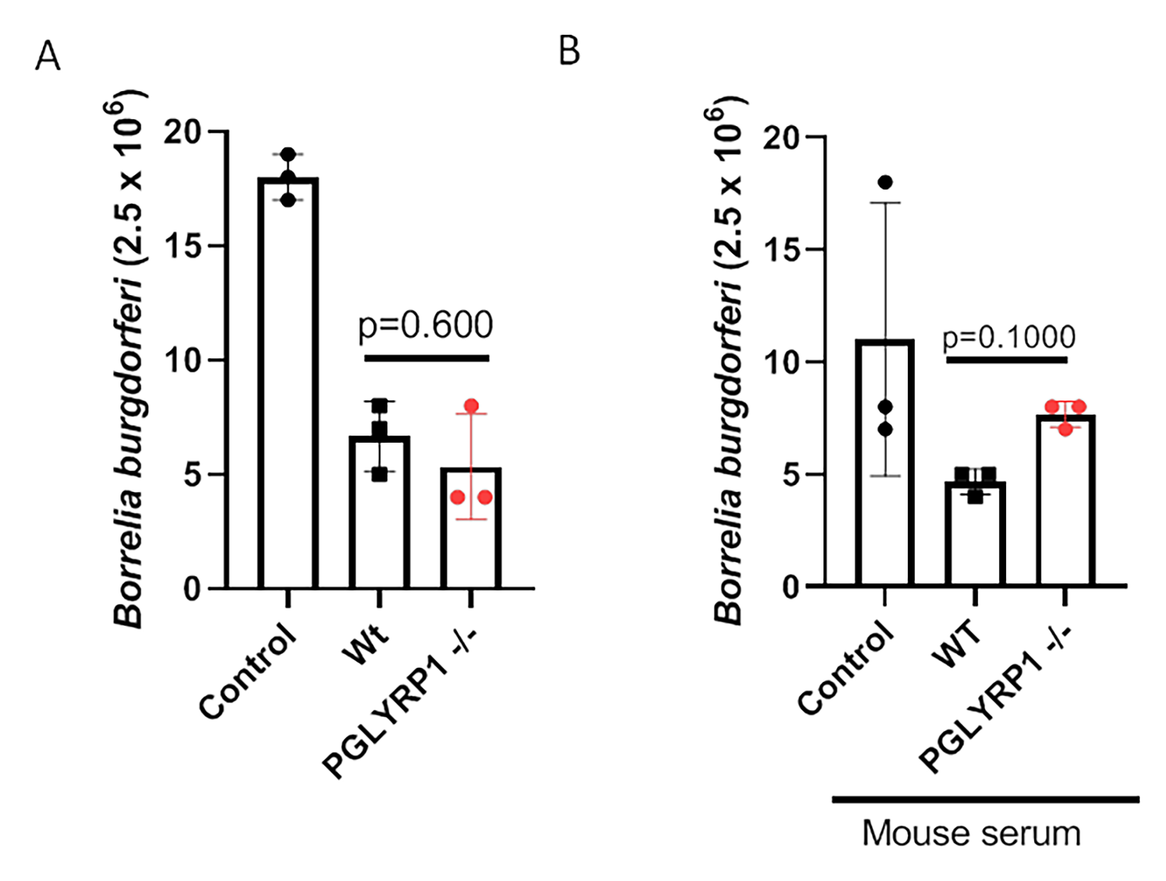

Supplement: S5 Fig — Neutrophils isolated from mouse bone marrow were incubated with 1x10^5^ B. burgdorferi for 1 hour in DMEM media in the absence (A) or presence of 10% mouse serum (B). The assay was performed in 96-well flat-bottom plates and volume was 50 μl. After 1 hour, 250 μl of BSK-H medium was added to the wells. The plates were incubated in microaerophilic conditions at 33°C for 72 hours. The Borrelia numbers were counted in Neubauer chamber under the dark-field microscope. The experiment was performed in triplicates and results from one independent experiment are shown. The bars represent mean ± SEM and p-values were determined by the Mann-Whitney test. (TIF) [file ppat.1009030.s008.tif]

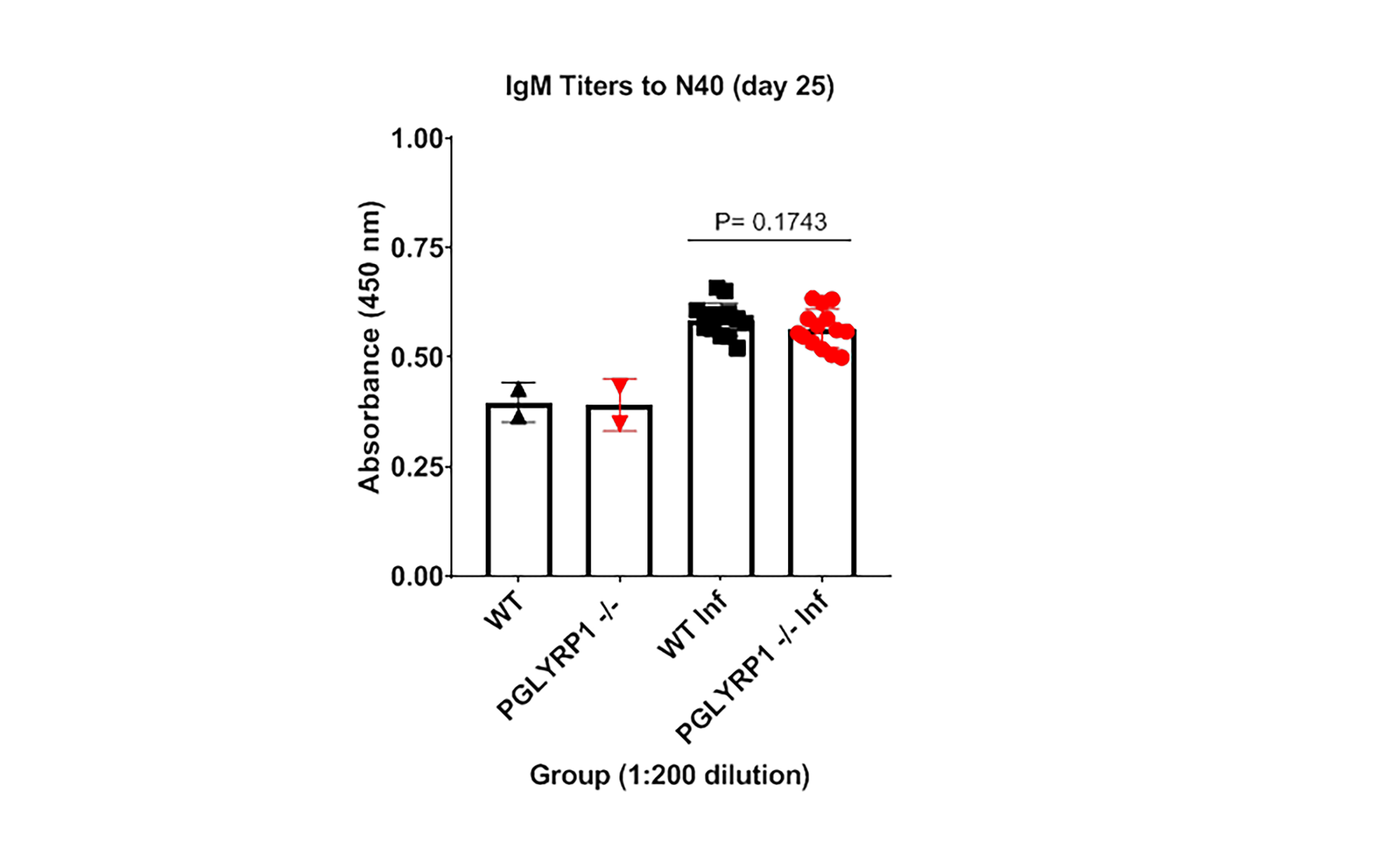

Supplement: S6 Fig — Antibody levels in uninfected wild type BALB/c (WT) and PGLYRP1-/- mice were compared with those in the infected ones (at least n = 7 in each group). Results from two independent experiment are shown. Whole-cell lysate of B. burgdorferi was coated on a microtiter plate and serum from either uninfected WT, infected WT, uninfected PGLYRP1-/- or infected PGLYRP1-/- mice was used at 1:200 dilution. The binding was measured by the secondary Goat anti-mouse IgM HRP-conjugated antibody. No significant difference in IgM level was observed in infected WT compared to infected PGLYRP1 knockout mice. Each data point represents an individual animal in the corresponding group. The bars represent mean ± SEM and p-values determined by student t-test. (TIF) [file ppat.1009030.s009.tif]

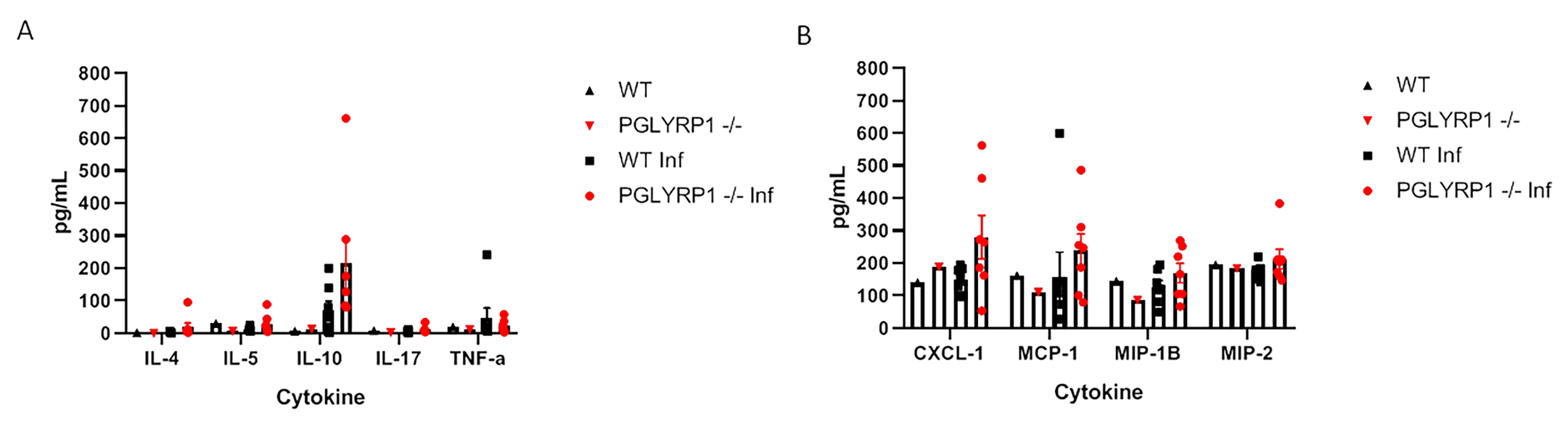

Supplement: S7 Fig — The infected PGLYRP1-/- mice also showed overall different levels (in pg/mL) of CXCL-1, MCP-1, MIP-1B, and MIP-2 (A) and TNF-α, IL-10, IL-4, IL-5, IL-10, IL-17 (B) as compared to BALB/c mice infected mice although the profiles were statistically insignificant. Representative results from one independent experiment are shown. Each data point represents an individual animal in the corresponding group. The bars represent mean ± SEM. (TIF) [file ppat.1009030.s010.tif]
